# Supplementary material for: Prevalence of physical health conditions and health risk behaviours in people with severe mental illness in South Asia: protocol for a cross-sectional study (IMPACT SMI survey)
Source: BMJ Open. 2020 Oct 10;10(10):e037869. doi: 10.1136/bmjopen-2020-037869 (PMC7549451; doi:10.1136/bmjopen-2020-037869)
Supplement: Author's manuscript [file bmjopen-2020-037869.draft_revisions.pdf]

BMJ Open

BMJ Open is committed to open peer review. As part of this commitment we make the peer review history of every article we publish publicly available.

When an article is published we post the peer reviewers' comments and the authors' responses online. We also post the versions of the paper that were used during peer review. These are the versions that the peer review comments apply to.

The versions of the paper that follow are the versions that were submitted during the peer review process. They are not the versions of record or the final published versions. They should not be cited or distributed as the published version of this manuscript.

BMJ Open is an open access journal and the full, final, typeset and author-corrected version of record of the manuscript is available on our site with no access controls, subscription charges or pay-per-view fees (http://bmjopen.bmj.com).

If you have any questions on BMJ Open's open peer review process please email info.bmjopen@bmj.com

BMJ Open

INVESTIGATING MENTAL AND PHYSICAL COMORBIDITY: Protocol for a survey in people with severe mental illness in South Asia (IMPACT SMI survey)

Journal:	*BMJ Open*
Manuscript ID	bmjopen-2020-037869
Article Type:	Protocol
Date Submitted by the Author:	19-Feb-2020

Complete List of Authors:	Zavala, Gerardo A.; University of York,<br>Prasad, Krishna; National Institute of Mental Health and Neuro Sciences<br>Aslam, Faiza; Institute of Psychiatry<br>Barua, Deepa; ARK Foundation<br>Haidar, Asiful; ARK Foundation<br>Hewitt, Catherine; The University of York, Health Sciences<br>Huque, Rumana; ARK Foundation<br>Mansoor, Sonia; Institute of Psychiatry<br>Murthy, Pratima; National Institute of Mental Health and NeuroScineces, Centre for Addiction Medicine<br>Nizami, Asad; Institute of Psychiatry<br>Siddiqi, Najma; University of York, Psychiatry, Hull York Medical School, York and Bradford District Care NHS Foundation Trust, Bradford, UK<br>Sikander, Siham; Health Services Academy<br>Siddiqi, Kamran; University of York, UK, Institute of Health Sciences<br>Boehnke, Jan Rasmus; University of Dundee, School of Nursing and Health Sciences

Keywords:	MENTAL HEALTH, Schizophrenia & psychotic disorders < PSYCHIATRY, PSYCHIATRY

SCHOLARONE™
Manuscripts

I, the Submitting Author has the right to grant and does grant on behalf of all authors of the Work (as defined in the below author licence), an exclusive licence and/or a non-exclusive licence for contributions from authors who are: i) UK Crown employees; ii) where BMJ has agreed a CC-BY licence shall apply, and/or iii) in accordance with the terms applicable for US Federal Government officers or employees acting as part of their official duties; on a worldwide, perpetual, irrevocable, royalty-free basis to BMJ Publishing Group Ltd ("BMJ") its licensees and where the relevant Journal is co-owned by BMJ to the co-owners of the Journal, to publish the Work in this journal and any other BMJ products and to exploit all rights, as set out in our licence.

The Submitting Author accepts and understands that any supply made under these terms is made by BMJ to the Submitting Author unless you are acting as an employee on behalf of your employer or a postgraduate student of an affiliated institution which is paying any applicable article publishing charge ("APC") for Open Access articles. Where the Submitting Author wishes to make the Work available on an Open Access basis (and intends to pay the relevant APC), the terms of reuse of such Open Access shall be governed by a Creative Commons licence – details of these licences and which Creative Commons licence will apply to this Work are set out in our licence referred to above.

Other than as permitted in any relevant BMJ Author's Self Archiving Policies, I confirm this Work has not been accepted for publication elsewhere, is not being considered for publication elsewhere and does not duplicate material already published. I confirm all authors consent to publication of this Work and authorise the granting of this licence.

For peer review only

INVESTIGATING MENTAL AND PHYSICAL COMORBIDITY: Protocol for a survey in people with severe mental illness in South Asia (IMPACT SMI survey)

G.A. Zavala [1], K. Prasad[2], F. Aslam[3], D. Barua[4], A. Haidar[4] C. Hewitt[1], R. Huque[4], S. Mansoor[3], P. Murthy[2], A. T. Nizami[3], N. Siddiqi[1,5,6], S. Sikander[7,8], K. Siddiqi[1,5], J.R. Boehnke[1,9] and on behalf of the IMPACT team.

Affiliations

[1]Department of Health Sciences, York University, Heslington, YO10 5DD, York, UK

[2] National Institute of Mental Health and Neurosciences (NIMHANS), Bangalore, India.

[3] Institute of Psychiatry (IoP), Rawalpindi, Pakistan

[4]ARK Foundation, Dhaka, Bangladesh

[5] Hull York Medical School, University of York Communications Office, Heslington, York, UK

[6] Bradford District Care NHS Foundation Trust, Bradford, UK

[7] Human Development Research Foundation, Islamabad, Pakistan

[8]Public Health, Health Services Academy, Islamabad, Pakistan

[9]School of Nursing and Health Sciences, University of Dundee, Dundee, UK

G.A. Zavala https://orcid.org/0000-0002-9825-8725 Corresponding author: gzavala@gmail.com

K. Prasad

F. Aslam https://orcid.org/0000-0002-7847-7250

D. Barua https://orcid.org/0000-0002-0122-9048

A. Haidar https://orcid.org/0000-0001-9015-4414

C. Hewitt https://orcid.org/0000-0002-0415-3536

R. Huque http://orcid.org/0000-0003-4724-0581

S. Mansoor http://orcid.org://0000-0002-9837-6852

P. Murthy https://orcid.org/0000-0002-7738-9670

A. T. Nizami https://orcid.org/0000-0002-7853-8519

N. Siddiqi https://orcid.org/0000-0003-1794-2152

S. Sikander https://orcid.org/0000-0002-0223-7234

K. Siddiqi https://orcid.org/0000-0003-1529-7778

J.R. Boehnke https://orcid.org/0000-0003-0249-1870

For peer review only

Abstract

Introduction: People with severe mental illness (SMI) die on average 10-20 years earlier than the general population. Most of these deaths are due to physical health conditions. The aim of this cross-sectional study is to determine the prevalence and determinants of physical disorders in people with SMI attending specialist mental health facilities in South Asia.

Methods: We will conduct a survey of patients with SMI attending specialist mental health facilities in Bangladesh, India and Pakistan (n=4,500). Diagnosis of SMI will be confirmed using the MINI V-6.0. We will collect information about: physical health and related health-risk behaviours (WHO STEPs); severity of common mental disorders (PHQ-9 and GAD-7); and health-related quality of life (EQ-5D-5L). We will measure blood pressure, height, weight and waist-circumference according to WHO guidelines. We will also measure glycated haemoglobin (HbA1c), lipid profile, thyroid function, liver function, creatinine and haemoglobin.

Analysis: Prevalence rates of physical health conditions and health-risk behaviours will be presented and compared with the WHO STEPs survey findings in the general population. Regression analyses will explore the association between health-risk behaviours, mental and physical health conditions. The IMPACT SMI survey will provide the prevalence of different physical health conditions and health-risk behaviours in the SMI population, and the influence of each of these conditions in terms of health related quality of life

Ethics and dissemination: The study has been approved by the ethics committees of the Department of Health Sciences University of York (UK), Centre for Injury Prevention and Rehabilitation (Bangladesh), Health Ministry Screening Committee and Indian Council of Medical Research (India) and National Bioethics Committee (Pakistan). Findings will be disseminated in peer reviewed articles, in local and international conferences and as reports for policy makers and stakeholders in the countries involved.

Trial registration: ISRCTN88485933; https://doi.org/10.1186/ISRCTN88485933, 3th of June 2019

Key words: Multimorbidity, Comorbidity, Lifestyle, Severe mental illness, South Asia, health risk behaviours, schizophrenia, bipolar disorders, psychosis

For peer review only - http://bmjopen.bmj.com/site/about/guidelines.xhtml

Article summary

Strengths and limitations

- The IMPACT SMI survey will provide the prevalence of different physical health conditions and health-risk behaviours in the SMI population in the South Asian region for the first time.
- The study is using standardized tools to measure patient outcomes that will allow us to compare the findings with those of the general population.
- The survey is being conducted in specialist centres (with patients with capacity) and is therefore a health care utilisation sample drawn largely from tertiary care.

For peer review only

For peer review only - http://bmjopen.bmj.com/site/about/guidelines.xhtml

Background

A considerable body of research has shown that people with severe mental illness (SMI; i.e. psychotic disorders, bipolar affective disorder and severe depression with psychosis) die on average 10-20 years earlier than the general population [1 2]. This excess mortality is a major global public health challenge but efforts to address it have been limited. Recent studies suggest that despite an overall improvement in life expectancy for both the general population and people with severe mental illness, the absolute mortality gap between these two groups is actually widening [3-5].

Around 80% of deaths in people with SMI are due to preventable physical illnesses, most commonly cardio-metabolic diseases, respiratory disorders, and infectious diseases [6 7]. Prevalence of most physical health conditions is higher and outcomes are poorer in this population.[8]. Multimorbidity (the presence of two or more conditions) is also more common [9] in the presence of SMI, contributing to poorer physical health and quality of life. The majority of evidence for these health inequalities has been generated in high-income countries (HICs), but a small number of studies from low- and middle-income countries (LMICs) also show a similar pattern of increased mortality for people with SMI, with an even shorter life expectancy, and larger mortality gap [7 10-12]. The few studies available suggest that physical comorbidity in SMI is at least as prevalent as in developed countries [1].

In South Asia, rates of mental illness and non-communicable diseases have been increasing rapidly [13 14]. This increase, coupled with the lack of access to even basic mental healthcare [15], and neglect of the physical health needs of people with SMI by policymakers and healthcare services[16], means that the burden of disease due to physical disorders in people with mental illness is set to rise further, with a corresponding increase in within country and global health inequalities. Addressing mental and physical health comorbidity in LMICs is a global priority, recognized in global policies to help achieve the Sustainable Development Goals, including "ensuring healthy lives and promoting well-being for all" [17-19]. A detailed understanding of the determinants of physical ill health in SMI in LMICs is needed to progress this agenda[20].

Objectives

In people with SMI attending specialist mental health facilities:

1. Determine the prevalence of physical disorders and health-risk behaviours and compare these findings with those established in the general population.
2. Determine the association between physical disorders, health-risk behaviours, health-related quality of life and various demographic, behavioural, cognitive, psychological and social variables.
3. Identify lifestyle advice that has been offered for this SMI population.
4. Support clinical trial readiness by providing evidence-based findings regarding outcome measures and procedures relevant for clinical trials design.

Methods and analysis

Design, settings & population

We will conduct a cross-sectional survey among patients with a clinical diagnosis of SMI recruited at specialist mental health institutions in Bangladesh (National Institute of Mental Health) (NIMH)), India (National Institute of Mental Health and Neurosciences (NIMHANS)) and Pakistan (Institute of Psychiatry (IoP)). All three are national institutes offering specialist mental health services. Adults (18 years and over) with a clinical diagnosis of SMI (i.e. schizophrenia, schizoaffective disorder, bipolar affective disorder, severe depression with psychosis) that are able to provide informed consent as assessed by the treating clinician will be recruited. Patients who lack capacity to provide consent or are unable to complete study questionnaires will be excluded (figure 1). The data will be collected from June 2019 until September 2020.

For peer review only - http://bmjopen.bmj.com/site/about/guidelines.xhtml

Figure 1. Flow diagram of the study

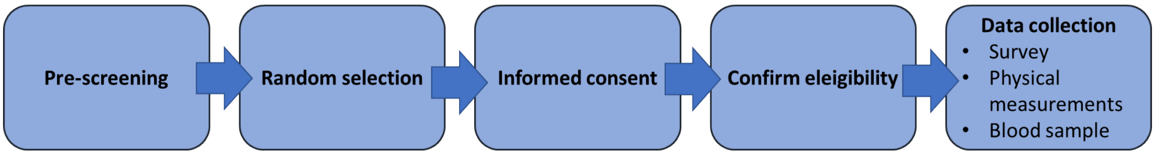

Sample size

We aim to build as large a sample as possible within the resources available over the study period (n=4500). As an indicative example of survey precision to address some of the key research questions, we provide a sample size estimate for investigating the prevalence of diabetes. Assuming a prevalence estimate of 10% (based on results from previous studies [21]), we will need 857 participants per country to achieve ± 2% precision assuming a 95% confidence interval[22].

Recruitment and random selection of the participants

Both in- and outpatients attending the specialist mental health institutions of the study will be recruited. When resources or the volume of patients do not allow recruitment of all patients presenting at the institution, we will use random selection of attending patients.

Informed consent

In the first instance, the researchers will give an information sheet to the patient, providing written and verbal information on the potential benefits and risks of taking part in the study and clarifying the assessments involved in the study. Only people who provided written informed consent will be included in the study. Where there are problems with literacy, the researchers will provide this information verbally to both the carer and the patient. Those unable to provide a signature will be requested to indicate their consent with a thumbprint. The information sheet will meet all the requirements of the relevant ethics committees. Maintenance of confidentiality and compliance with international, UK and relevant local Data Protection Acts will be emphasised to all study participants. Moreover, all participants will be informed that their participation in the study is voluntary, consent can be withdrawn at any stage and the decision about participation will not affect their care.

Mental capacity is time- and decision-specific. For patients who are assessed to lack capacity by their local physician, seeking informed consent will be delayed until capacity is regained, if feasible. No assessments will be continued where the patient appears reluctant, even where patient consent has been obtained.

Optional 'consent to contact' about future studies will be sought from survey participants. Permission will be sought to retain participant details and to contact participants about future studies that may be relevant for them. Again, it will be emphasised that this is voluntary, they can withdraw permission at any time and this will not affect their care in any way. After consenting to participate, the participant will be given a unique patient identifier (ID) by the researcher. The patient consent and contact information will be entered into a database (participant log), which will be kept separate from the survey results database. The survey results database will include only the unique patient ID, and no patient identifiable data.

Confirmation of eligibility

SMI diagnosis will be confirmed by trained researchers using the relevant modules from the Mini-international neuropsychiatric interview (MINI) version 6.0 [23]. The MINI is a short diagnostic structured interview to explore mental disorders. It is designed to allow administration by non-specialist interviewers[24]. The inter-rater reliability for the MINI will be assessed at each site at the start of study. Within each study site, at least 10 MINI questionnaires will be completed and compared between two different researchers blinded from each other. Where needed, further training will be offered to achieve acceptable inter-rater reliability before researchers are assigned to the study.

For peer review only

For peer review only - http://bmjopen.bmj.com/site/about/guidelines.xhtml

Data collection

We will conduct a face-to-face digital survey to collect information about physical health and health-risk behaviours, severity of common mental disorders and health-related quality of life using validated instruments as described below.

The WHO STEPwise approach to Surveillance (STEPs) instrument Version 3.2 will be used to collect information about non-communicable diseases and related health-risk behaviours[25]. STEPs is an international standardized tool that will allow comparisons with the general population within the country and between countries[26]. The instrument has already been translated, used and validated in the general population in Bangladesh, India and Pakistan [27-29].

To allow patients to disclose information on sexually transmitted diseases, HIV diagnosis, alcohol and tobacco consumption, in agreement with patient's wishes, the interviewer will ask the caregivers/ attendants to leave the room for this part of the interview

Demographic information

The STEPs demographic module will be used to collect information on age, sex, education, marital status, employment and income [25].

Health-risk behaviours

The STEPs tobacco modules will be used to assess tobacco-related behaviours [25].

Alcohol consumption

The STEPs alcohol module will be used to determine lifetime abstainers, past 12 months abstainers and current users of alcohol using the WHO cut-off scores [25].

Diet

The STEPs diet module will be used to record the number of days that respondents consumed fruit and vegetables in a typical week, and the number of servings of fruit and vegetables consumed on average per day [30].

Physical activity

The STEPs physical activity module will be used to record activity for transport purposes (e.g. walking, cycling), vigorous and moderate activity at work, and vigorous, moderate activity in leisure time, and time spent sitting. Show-cards with culturally relevant examples will be used to aid respondents in classifying activities. Analysis and categorization will follow the WHO guidelines[31].

Non-communicable diseases

Any medically-diagnosed history of raised blood pressure, heart disease, hypercholesterolemia, and high blood glucose, and treatment advised by a health worker for these diseases (such as prescribed medicines, a special diet, advice to reduce salt intake, lose weight, stop smoking, or do more exercise) will be collected using the STEPs module for non-communicable diseases. Lung disease is not included in the STEPSs survey. However, it will be recorded asking the same set of questions as the non-communicable diseases included in the STEPs survey.

Infectious diseases

Self-report of medically-diagnosed history of hepatitis B, C, syphilis, tuberculosis, HIV and intestinal parasites will be recorded.

Risk behaviours related to sexually transmitted diseases

Risk behaviours related to sexually transmitted diseases will be assessed using three questions that examine the presence of multiple sexual partners, unprotected sexual contact and use of injectable drugs that have been adapted from the 10 item HIV risk Screening Instrument [32]. This instrument has been previously used in research involving persons with severe mental illness in India [33].

For peer review only - http://bmjopen.bmj.com/site/about/guidelines.xhtml

Depressive and anxiety symptoms

The 9-item depression module (PHQ-9)[34] will be used to measure the severity of depressive symptoms and the generalized anxiety disorder 7 item scale (GAD-7)[35] for the severity of anxiety symptoms.

Health-Related Quality of life

The EQ-5D-5L will be used to measure health-related quality of life [36 37]. The EQ-5D-5L is a standardised measure of health status, it provides a simple, generic measure of health for clinical and economic appraisal. The impact of disease/disorder is characterised on five dimensions (mobility, self-care, ability to undertake usual activities, pain, anxiety / depression). The EQ-5D-5L further assesses a patient's subjective evaluation of their health state based on a visual analogue scale (EQ-5d-VAS) between "0 - worst imaginable health state" and "100 - best imaginable health state".

Blood pressure and heart rate

Blood pressure (BP) and pulse rate will be taken three times using an automated blood pressure measuring instrument (OMRON®). For BP measurement the participant must be comfortable and rested. If he/she had been exerting themselves, then there will be a minimum of a 15 minute rest period before the recording is taken. There should be at least a three minute gap between the BP recordings. The procedure will follow the step wise instructions in the WHO STEPS surveillance manual[38].

Height weight and waist circumference

Height will be measured to a precision of 0.1 cm using a portable height measuring board without footwear and headgear. Weight will be measured in kilograms using a portable weighing scale placed on a firm flat surface with light clothing and without footwear and socks. Waist circumference will be measured in to a precision of 0.1 cm, using a flexible fibre glass anthropometric tape at the end of normal expiration, at the midpoint between the lower margin of the last palpable rib and the top of the iliac crest (hip bone), with the arms relaxed at the sides. All measurements will be taken in duplicate and the average of the two values will be recorded and the protocols of the WHO STEPS surveillance manual will be followed[38].

Biochemical analysis

A blood sample (8 to 9 ml) will be taken from consenting participants for: glycated haemoglobin (HbA1c), lipid profile (triglycerides, total cholesterol, HDL cholesterol, LDL cholesterol), thyroid function tests (includes T3, T4, TSH), liver function tests (total Bilirubin, AST, ALT, ALP, total protein, albumin, albumin to globulin ratio), creatinine and haemoglobin.

Data management

To ensure quality of collected data, validation, checking, proofing and cleaning procedures will be carried out according to standard procedures[39]. All consent forms will be stored separately from survey data in locked cabinets in locked offices at study research offices at each study site. All coded data will be transferred to, and stored as anonymous data at the University of York which will act as the data curator. A secure password protected and encrypted electronic database will be set up to store the data.

Statistical analysis

Quantitative data will be summarised using descriptive statistics. First, prevalence rates of infectious diseases, long-term physical health conditions (e.g. diabetes, heart disease) and health-risk behaviours for type II diabetes, cardiovascular and respiratory disorders (e.g. tobacco use, obesity, physical activity, diet) will be estimated in the severe mental illness population. Second, these rates will be compared with results from the WHO STEPs survey in the general population. Finally, we will examine the associations between physical health conditions and health-risk behaviours, using regression models with random intercepts for data collection site with the following potential covariates: demographic variables (i.e. age, sex, and level of education), history of mental and physical conditions and biomarkers (e.g. BMI, blood pressure). These regression models will also be used to calculate prevalence rates of physical health conditions and health-risk behaviors adjusted for these covariates.

For peer review only - http://bmjopen.bmj.com/site/about/guidelines.xhtml

Further regression analyses will explore the association between mental and physical health conditions and health-related quality of life measured using EQ-5D-5L.

Regression models will take account of the nature and distribution of data (e.g. count versus continuous data; skewed versus normally distributed data). Additional analyses using network models [40] and psychometric approaches will investigate comorbidity patterns (within and across physical and mental symptoms) as well as the structure of severe mental illness symptoms[41-43].

Ethics and dissemination

The study has been approved by the ethics committees of the Department of Health Sciences University of York (UK), Centre for Injury Prevention and Rehabilitation (Bangladesh), Health Ministry Screening Committee and Indian Council of Medical Research (India) and National Bioethics Committee (Pakistan).

The study will adhere to the fundamental principles of human rights and dignity laid down in the Declaration of Helsinki[44]. Study procedures will comply with legislation and guidance for good practice governing the participation in research of people lacking capacity as set out in the Mental Capacity Act (UK) 2005[45]. Participants will not receive any financial inducement to participate, but the results of the physical measurements and blood tests will be shared with the participant and the treating clinician.

As a non-interventional study, there is minimal risk of adverse events associated with the study. Due to the vulnerability of the severe mental illness population, however, there is a potential (albeit low) risk that some questions or the burden of the assessments may cause distress for participants or the family carers. If there is any indication of this, the survey or assessments will be stopped, the participant offered reassurance and support, and if needed, referral to the clinical team. If a participant during the interview reveals any suicidal ideation, the interviewer will refer them to the treating clinician for a clinical risk assessment and further management. If a physical condition or blood test abnormality (outside the normal range for age and sex) is detected as a part of the research assessments, the research team will inform the clinician responsible for the patient, who will evaluate the result and give the patient the option of referral to a specialist if this is required for their condition.

Community, patient and public involvement

A ‘Community Panel’ comprising stakeholders from these constituencies ensures community, patient and public involvement. The Panel has reviewed the planned survey and advised on its feasibility. The panel will continue to provide feedback on the conduct of the study and presentation of findings. To support dissemination efforts, findings will be presented to the community panel, with advice sought about the format and content of lay summaries and other outputs aimed at patients and the public.

Dissemination

Findings will be disseminated in peer-reviewed articles, in local and international conferences and as reports for policy makers and stakeholders in the countries involved.

Discussion

People with SMI have a reduced life expectancy. Research in high income countries has shown that a large proportion of this inequality is accounted for by chronic diseases [1 2]. This has been consistently observed in high income countries [6 7]. However information is lacking in minority groups and LMICs, which is surprising considering the increased risk of physical health conditions in these populations due to poverty, education, poor diet, low access to healthcare, exposure to infectious diseases and other environmental factors [46]. The IMPACT SMI survey will address this gap in knowledge, demonstrating the prevalence of different physical health conditions and health-risk behaviours in the SMI population, and the influence of each of these conditions in terms of health related quality of life. It will support SMI clinical trial readiness by providing evidence-based findings regarding outcome measures and procedures relevant for clinical trials design.

For peer review only - http://bmjopen.bmj.com/site/about/guidelines.xhtml

Strengths and limitations

To the best of the authors' knowledge, this will be the first large scale study to investigate the physical health and health-risk behaviours in the SMI population in LMICs. The main limitation is that the survey is conducted in specialist centres (with patients with capacity) and is therefore a health care utilisation sample drawn largely from tertiary care. The gaps in treatment and care utilisation and systematic factors influencing these have been well documented (e.g. [47]), therefore, the study population may not be representative of the total severe mental illness population in each country. However, a community survey would not be feasible, and primary and secondary care mental health services are not sufficiently developed to be used to recruit participants for this survey (see for example work by PRIME [15] and Emerald [48] programmes on strengthening these services as access points). However, unlike services in high-income countries, tertiary care services in these South Asian countries accept self-referral without the need for primary or secondary care referral and we include outpatient services offered by the collaborating centres which often function as 'the first port of call' for people with mental illness[49-51]. Therefore, the selected centres are likely covering a broad range of the treatment-seeking population.

Conclusion

Multimorbidity in people with SMI is not well documented particularly in low- and middle-income countries. The IMPACT survey will be the first to study it using a standardised approach in South Asia that can be compared to the general population.

Acknowledgements

We would like to acknowledge Dr Sue Bellass, Prof. Santosh Kumar Chaturvedi, Dr Arun Kandasamy, Dr Noreen Mdege, Dr Masuma Mishu, Dr Emily Peckham, Prof. Simon Gilbody and the research teams at ARK foundation and The National Institute of Mental Health (NIMH) in Bangladesh , Institute of Psychiatry in Pakistan (IoP) and The National Institute of Mental Health and Neuro-Sciences (NIMHANS) in India for their contribution to the study. We want to thank all the participants and caregivers for taking the time to complete the survey.

References

1. Liu NH, Daumit GL, Dua T, et al. Excess mortality in persons with severe mental disorders: a multilevel intervention framework and priorities for clinical practice, policy and research agendas. *World psychiatry : official journal of the World Psychiatric Association (WPA)* 2017;16(1):30-40. doi: 10.1002/wps.20384 [published Online First: 2017/01/28]

2. Walker ER, McGee RE, Druss BG. Mortality in mental disorders and global disease burden implications: a systematic review and meta-analysis. *JAMA psychiatry* 2015;72(4):334-41. doi: 10.1001/jamapsychiatry.2014.2502 [published Online First: 2015/02/12]

3. Olfson M, Gerhard T, Huang C, et al. Premature Mortality Among Adults With Schizophrenia in the United States. *JAMA psychiatry* 2015;72(12):1172-81. doi: 10.1001/jamapsychiatry.2015.1737 [published Online First: 2015/10/29]

4. Saha S, Chant D, McGrath J. A systematic review of mortality in schizophrenia: is the differential mortality gap worsening over time? *Archives of general psychiatry* 2007;64(10):1123-31. doi: 10.1001/archpsyc.64.10.1123 [published Online First: 2007/10/03]

5. Chesney E, Goodwin GM, Fazel S. Risks of all-cause and suicide mortality in mental disorders: a meta-review. *World psychiatry : official journal of the World Psychiatric Association (WPA)* 2014;13(2):153-60. doi: 10.1002/wps.20128 [published Online First: 2014/06/04]

6. Laursen TM. Life expectancy among persons with schizophrenia or bipolar affective disorder. *Schizophrenia research* 2011;131(1-3):101-4. doi: 10.1016/j.schres.2011.06.008 [published Online First: 2011/07/12]

7. Fekadu A, Medhin G, Kebede D, et al. Excess mortality in severe mental illness: 10-year population-based cohort study in rural Ethiopia. *The British journal of psychiatry : the journal of mental science* 2015;206(4):289-96. doi: 10.1192/bjp.bp.114.149112 [published Online First: 2015/02/07]

For peer review only - http://bmjopen.bmj.com/site/about/guidelines.xhtml

8. Mai Q, Holman CD, Sanfilippo FM, et al. The impact of mental illness on potentially preventable hospitalisations: a population-based cohort study. *BMC psychiatry* 2011;11:163. doi: 10.1186/1471-244X-11-163 [published Online First: 2011/10/12]

9. Reilly S, Olier I, Planner C, et al. Inequalities in physical comorbidity: a longitudinal comparative cohort study of people with severe mental illness in the UK. *BMJ open* 2015;5(12):e009010. doi: 10.1136/bmjopen-2015-009010 [published Online First: 2015/12/17]

10. Kurihara T, Kato M, Reverger R, et al. Seventeen-year clinical outcome of schizophrenia in Bali. *European psychiatry : the journal of the Association of European Psychiatrists* 2011;26(5):333-8. doi: 10.1016/j.eurpsy.2011.04.003 [published Online First: 2011/06/24]

11. Mogga S, Prince M, Alem A, et al. Outcome of major depression in Ethiopia: population-based study. *The British journal of psychiatry : the journal of mental science* 2006;189:241-6. doi: 10.1192/bjp.bp.105.013417 [published Online First: 2006/09/02]

12. Teferra S, Shibre T, Fekadu A, et al. Five-year mortality in a cohort of people with schizophrenia in Ethiopia. *BMC psychiatry* 2011;11:165. doi: 10.1186/1471-244x-11-165 [published Online First: 2011/10/12]

13. Nambiar D, Razzak J, Afsana K, et al. Mental illness and injuries: emerging health challenges of urbanisation in South Asia. *bmj* 2017;357:j1126.

14. Misra A, Tandon N, Ebrahim S, et al. Diabetes, cardiovascular disease, and chronic kidney disease in South Asia: current status and future directions. *bmj* 2017;357:j1420.

15. Lund C, Tomlinson M, Patel V. Integration of mental health into primary care in low-and middle-income countries: the PRIME mental healthcare plans. *The British Journal of Psychiatry* 2016;208(s56):s1-s3.

16. Shavitt S, Cho YI, Johnson TP, et al. Culture moderates the relation between perceived stress, social support, and mental and physical health. *Journal of Cross-Cultural Psychology* 2016;47(7):956-80.

17. Collins PY, Insel TR, Chockalingam A, et al. Grand challenges in global mental health: integration in research, policy, and practice. *PLoS Med* 2013;10(4):e1001434. doi: 10.1371/journal.pmed.1001434 [published Online First: 2013/05/03]

18. Baingana F, al'Absi M, Becker AE, et al. Global research challenges and opportunities for mental health and substance-use disorders. *Nature* 2015;527(7578):S172-7. doi: 10.1038/nature16032 [published Online First: 2015/11/19]

19. UN. Transforming our world: the 2030 Agenda for Sustainable Development. New York: United Nations, 2015.

20. Stubbs B, Koyanagi A, Veronese N, et al. Physical multimorbidity and psychosis: comprehensive cross sectional analysis including 242,952 people across 48 low-and middle-income countries. *BMC medicine* 2016;14(1):189.

21. Vancampfort D, Correll CU, Galling B, et al. Diabetes mellitus in people with schizophrenia, bipolar disorder and major depressive disorder: a systematic review and large scale meta-analysis. *World psychiatry : official journal of the World Psychiatric Association (WPA)* 2016;15(2):166-74. doi: 10.1002/wps.20309 [published Online First: 2016/06/07]

22. Buderer NMF. Statistical methodology: I. Incorporating the prevalence of disease into the sample size calculation for sensitivity and specificity. *Academic Emergency Medicine* 1996;3(9):895-900.

23. Lecrubier Y, Sheehan DV, Weiller E, et al. The Mini International Neuropsychiatric Interview (MINI). A short diagnostic structured interview: reliability and validity according to the CIDI. *European Psychiatry* 1997;12(5):224-31. doi: https://doi.org/10.1016/S0924-9338(97)83296-8

24. Sheehan DV, Lecrubier Y, Harnett Sheehan K, et al. The validity of the Mini International Neuropsychiatric Interview (MINI) according to the SCID-P and its reliability. *European Psychiatry* 1997;12(5):232-41. doi: https://doi.org/10.1016/S0924-9338(97)83297-X

For peer review only - http://bmjopen.bmj.com/site/about/guidelines.xhtml

25. Bonita R, Winkelmann R, Douglas KA, et al. The WHO Stepwise approach to surveillance (STEPS) of non-communicable disease risk factors. Global behavioral risk factor surveillance: Springer 2003:9-22.

26. Bonita R, De Courten M, Dwyer T, et al. Surveillance of risk factors for noncommunicable diseases: the WHO STEPwise approach: summary: Noncommunicable Diseases and Mental Health, World Health Organization, 2001.

27. Nishtar S, Bile KM, Ahmed A, et al. Integrated population-based surveillance of noncommunicable diseases: the Pakistan model. *American journal of preventive medicine* 2005;29(5):102-06.

28. Mohan V, Mathur P, Deepa R, et al. Urban rural differences in prevalence of self-reported diabetes in India—The WHO–ICMR Indian NCD risk factor surveillance. *Diabetes research and clinical practice* 2008;80(1):159-68.

29. Zaman MM, Rahman MM, Rahman MR, et al. Prevalence of risk factors for non-communicable diseases in Bangladesh: results from STEPS survey 2010. *Indian J Public Health* 2016;60(1):17-25.

30. Fulton SL, McKinley MC, Young IS, et al. The effect of increasing fruit and vegetable consumption on overall diet: a systematic review and meta-analysis. *Critical reviews in food science and nutrition* 2016;56(5):802-16.

31. Cleland CL, Hunter RF, Kee F, et al. Validity of the global physical activity questionnaire (GPAQ) in assessing levels and change in moderate-vigorous physical activity and sedentary behaviour. *BMC public health* 2014;14(1):1255.

32. Gerbert B, Bronstone A, McPhee S, et al. Development and testing of an HIV-risk screening instrument for use in health care settings. *American Journal of Preventive Medicine* 1998;15(2):103-13.

33. Chandra PS, Carey MP, Carey KB, et al. HIV risk behaviour among psychiatric inpatients: results from a hospital-wide screening study in southern India. *International journal of STD & AIDS* 2003;14(8):532-38.

34. Kroenke K, Spitzer RL. The PHQ-9: a new depression diagnostic and severity measure. *Psychiatric annals* 2002;32(9):509-15.

35. Löwe B, Decker O, Müller S, et al. Validation and standardization of the Generalized Anxiety Disorder Screener (GAD-7) in the general population. *Medical care* 2008;46(3):266-74.

36. Hurst NP, Kind P, Ruta D, et al. Measuring health-related quality of life in rheumatoid arthritis: validity, responsiveness and reliability of EuroQol (EQ-5D). *British journal of rheumatology* 1997;36(5):551-9.

37. EuroQol Group. EQ-5D-5L User Guide: Basic information on how to use the EQ-5D-5L instrument (version 2.0) 2013 04/03/2015. 1990. http://www.euroqol.org/fileadmin/user_upload/Documenten/PDF/Folders_Flyers/UserGuide_EQ-5D-5L_v2.0_October_2013.pdf. (accessed 09/05/2017).

38. Organization WH. WHO STEPS surveillance manual: the WHO STEPwise approach to chronic disease risk factor surveillance: Geneva: World Health Organization, 2005.

39. Corti L, Van den Eynden V, Bishop L, et al. Managing and sharing research data: a guide to good practice: Sage 2014.

40. Kossakowski JJ, Epskamp S, Kieffer JM, et al. The application of a network approach to Health-Related Quality of Life (HRQoL): introducing a new method for assessing HRQoL in healthy adults and cancer patients. *Quality of Life Research* 2016;25(4):781-92.

41. Böhnke J, Croudace TJ. Factors of psychological distress: clinical value, measurement substance, and methodological artefacts. *Social psychiatry and psychiatric epidemiology* 2015;50(4):515-24.

42. Eaton NR, Rodriguez-Seijas C, Carragher N, et al. Transdiagnostic factors of psychopathology and substance use disorders: a review. *Social psychiatry and psychiatric epidemiology* 2015;50(2):171-82.

43. Goldberg D. Psychopathology and classification in psychiatry. *Social psychiatry and psychiatric epidemiology* 2015;50(1):1-5.

For peer review only

44. Williams JR. The Declaration of Helsinki and public health. *Bulletin of the World Health Organization* 2008;86:650-52.

45. Boyle G. The Mental Capacity Act 2005: promoting the citizenship of people with dementia? *Health & social care in the community* 2008;16(5):529-37.

46. Allen L, Williams J, Townsend N, et al. Socioeconomic status and non-communicable disease behavioural risk factors in low-income and lower-middle-income countries: a systematic review. *The Lancet Global Health* 2017;5(3):e277-e89.

47. Evans-Lacko S, Aguilar-Gaxiola S, Al-Hamzawi A, et al. Socio-economic variations in the mental health treatment gap for people with anxiety, mood, and substance use disorders: results from the WHO World Mental Health (WMH) surveys. *Psychol Med* 2018;48(9):1560-71. doi: 10.1017/s0033291717003336 [published Online First: 2017/11/28]

48. Semrau M, Evans-Lacko S, Alem A, et al. Strengthening mental health systems in low-and middle-income countries: the Emerald programme. *BMC medicine* 2015;13(1):79.

49. Afsar HA, Younus M. Patient referral at the grass-roots level in Pakistan. *Nat Sci(Internet)* 2004;2(4):18-27.

50. Karim S, Saeed K, Rana MH, et al. Pakistan mental health country profile. *International Review of Psychiatry* 2004;16(1-2):83-92.

51. Organization WH. Bangladesh health system review: Manila: WHO Regional Office for the Western Pacific 2015.

Authors' contributions

NS conceived the study, KP, FA, DB, RH, SM, PM, AN, SK and KS provided important contextual information, KP, FP, DB, RH, SM, NS and JB developed the methodology and selected the outcomes, GZ, AH, KH and JB developed the analysis plan, GZ, NS, KS and JB wrote the manuscript, and all authors revised and approved the manuscript.

Funding statement

This research was funded by the National Institute for Health Research (NIHR) (17/63/130) using UK aid from the UK Government to support global health research. The views expressed in this publication are those of the author(s) and not necessarily those of the NIHR or the UK Department of Health and Social Care. The funders had no role in study design, data collection and analysis, decision to publish, or preparation of the manuscript.

Competing interests statement

The authors declare that they have no competing interests

Word count

3159 words

For peer review only - http://bmjopen.bmj.com/site/about/guidelines.xhtml

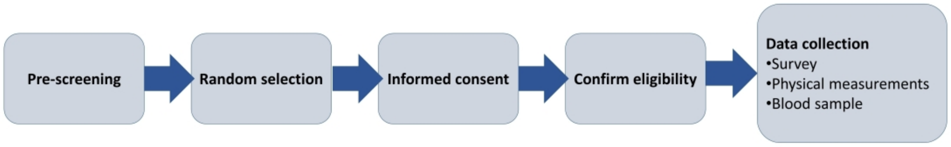

Figure 1. Flow diagram of the study

1399x600mm (96 x 96 DPI)

INVESTIGATING MENTAL AND PHYSICAL COMORBIDITY: Protocol for a survey in people with severe mental illness in South Asia (IMPACT SMI survey)

STROBE Statement—Checklist of items that should be included in reports of ***cross-sectional studies***

	Item No	Recommendation
**Title and abstract**	1	(*a*) Indicate the study's design with a commonly used term in the title or the abstract<br>**Title and abstract lines 1 to 56**
		(*b*) Provide in the abstract an informative and balanced summary of what was done and what was found<br>**Abstract lines 32 to 56**
**Introduction**		
Background/rationale	2	Explain the scientific background and rationale for the investigation being reported<br>**Background paragraphs 1 to 3**
Objectives	3	State specific objectives, including any prespecified hypotheses<br>**Background, objectives section lines 93 to 101**
**Methods**		
Study design	4	Present key elements of study design early in the paper<br>**Methods paragraph 1 design setting and population 103 to 115**
Setting	5	Describe the setting, locations, and relevant dates, including periods of recruitment, exposure, follow-up, and data collection<br>**Methods paragraph 1 design setting and population lines 103 to 115**
Participants	6	(*a*) Give the eligibility criteria, and the sources and methods of selection of participants<br>**Methods paragraph 1 design setting and population lines 103 to 115**
Variables	7	Clearly define all outcomes, exposures, predictors, potential confounders, and effect modifiers. Give diagnostic criteria, if applicable<br>**Methods, data collection section lines 158 to 231**
Data sources/ measurement	8*	For each variable of interest, give sources of data and details of methods of assessment (measurement). Describe comparability of assessment methods if there is more than one group<br>**Methods, data collection section lines 158 to 231**
Bias	9	Describe any efforts to address potential sources of bias<br>**Discussion, strengths and limitation section lines 297 to 307**
Study size	10	Explain how the study size was arrived at<br>**Methods section Sample size lines 298 to 311**
Quantitative variables	11	Explain how quantitative variables were handled in the analyses. If applicable, describe which groupings were chosen and why<br>**NA**
Statistical methods	12	(*a*) Describe all statistical methods, including those used to control for confounding<br>**Methods Statistical analysis section lines 240 to 255**
		(*b*) Describe any methods used to examine subgroups and interactions<br>**Methods Statistical analysis section lines 240 to 255**
		(*c*) Explain how missing data were addressed<br>**NA**
		(*d*) If applicable, describe analytical methods taking account of sampling strategy<br>**Methods, Recruitment and random selection of the participants lines 123 to 126**
		(*e*) Describe any sensitivity analyses

For peer review only - http://bmjopen.bmj.com/site/about/guidelines.xhtml

		**Methods Statistical analysis section lines 239 to 255**
**Results**		
Participants	13*	(a) Report numbers of individuals at each stage of study—eg numbers potentially eligible, examined for eligibility, confirmed eligible, included in the study, completing follow-up, and analysed<br>**NA**
		(b) Give reasons for non-participation at each stage<br>NA
		(c) Consider use of a flow diagram<br>**NA**
Descriptive data	14*	(a) Give characteristics of study participants (eg demographic, clinical, social) and information on exposures and potential confounders
		(b) Indicate number of participants with missing data for each variable of interest<br>**NA**
Outcome data	15*	Report numbers of outcome events or summary measures<br>**NA**
Main results	16	(*a*) Give unadjusted estimates and, if applicable, confounder-adjusted estimates and their precision (eg, 95% confidence interval). Make clear which confounders were adjusted for and why they were included
		(*b*) Report category boundaries when continuous variables were categorized
		(b) If relevant, consider translating estimates of relative risk into absolute risk for a meaningful time period<br>**NA**
Other analyses	17	Report other analyses done—eg analyses of subgroups and interactions, and sensitivity analyses<br>**NA**
**Discussion**		
Key results	18	Summarise key results with reference to study objectives<br>**NA**
Limitations	19	Discuss limitations of the study, taking into account sources of potential bias or imprecision. Discuss both direction and magnitude of any potential bias<br>**NA**
Interpretation	20	Give a cautious overall interpretation of results considering objectives, limitations, multiplicity of analyses, results from similar studies, and other relevant evidence<br>**NA**
Generalisability	21	Discuss the generalisability (external validity) of the study results<br>**NA**
**Other information**		
Funding	22	Give the source of funding and the role of the funders for the present study and, if applicable, for the original study on which the present article is based<br>**NA**

*Give information separately for exposed and unexposed groups.

For peer review only

Note: An Explanation and Elaboration article discusses each checklist item and gives methodological background and published examples of transparent reporting. The STROBE checklist is best used in conjunction with this article (freely available on the Web sites of PLoS Medicine at http://www.plosmedicine.org/, Annals of Internal Medicine at http://www.annals.org/, and Epidemiology at http://www.epidem.com/). Information on the STROBE Initiative is available at www.strobe-statement.org.

For peer review only

For peer review only - http://bmjopen.bmj.com/site/about/guidelines.xhtml

BMJ Open

Prevalence of physical health conditions and health risk behaviours in people with severe mental Illness in South Asia: Protocol for a cross-sectional study (IMPACT SMI survey)

Journal:	*BMJ Open*
Manuscript ID	bmjopen-2020-037869.R1
Article Type:	Protocol
Date Submitted by the Author:	13-Jun-2020
Complete List of Authors:	Zavala, Gerardo A.; University of York,<br>Prasad, Krishna; National Institute of Mental Health and Neuro Sciences<br>Aslam, Faiza; Institute of Psychiatry<br>Barua, Deepa; ARK Foundation<br>Haidar, Asiful; ARK Foundation<br>Hewitt, Catherine; The University of York, Health Sciences<br>Huque, Rumana; ARK Foundation<br>Mansoor, Sonia; Institute of Psychiatry<br>Murthy, Pratima; National Institute of Mental Health and NeuroScineces, Centre for Addiction Medicine<br>Nizami, Asad; Institute of Psychiatry<br>Siddiqi, Najma; University of York, Psychiatry, Hull York Medical School, York and Bradford District Care NHS Foundation Trust, Bradford, UK<br>Sikander, Siham; Health Services Academy<br>Siddiqi, Kamran; University of York, UK, Institute of Health Sciences<br>Boehnke, Jan Rasmus; University of Dundee, School of Nursing and Health Sciences
**Primary Subject Heading**:	Global health
Secondary Subject Heading:	Global health, Mental health, Epidemiology, Public health
Keywords:	MENTAL HEALTH, Schizophrenia & psychotic disorders < PSYCHIATRY, PSYCHIATRY

SCHOLARONE™
Manuscripts

I, the Submitting Author has the right to grant and does grant on behalf of all authors of the Work (as defined in the below author licence), an exclusive licence and/or a non-exclusive licence for contributions from authors who are: i) UK Crown employees; ii) where BMJ has agreed a CC-BY licence shall apply, and/or iii) in accordance with the terms applicable for US Federal Government officers or employees acting as part of their official duties; on a worldwide, perpetual, irrevocable, royalty-free basis to BMJ Publishing Group Ltd ("BMJ") its licensees and where the relevant Journal is co-owned by BMJ to the co-owners of the Journal, to publish the Work in this journal and any other BMJ products and to exploit all rights, as set out in our licence.

The Submitting Author accepts and understands that any supply made under these terms is made by BMJ to the Submitting Author unless you are acting as an employee on behalf of your employer or a postgraduate student of an affiliated institution which is paying any applicable article publishing charge ("APC") for Open Access articles. Where the Submitting Author wishes to make the Work available on an Open Access basis (and intends to pay the relevant APC), the terms of reuse of such Open Access shall be governed by a Creative Commons licence – details of these licences and which Creative Commons licence will apply to this Work are set out in our licence referred to above.

Other than as permitted in any relevant BMJ Author's Self Archiving Policies, I confirm this Work has not been accepted for publication elsewhere, is not being considered for publication elsewhere and does not duplicate material already published. I confirm all authors consent to publication of this Work and authorise the granting of this licence.

For peer review only

Prevalence of physical health conditions and health risk behaviours in people with severe mental Illness in South Asia: Protocol for a cross-sectional study (IMPACT SMI survey)

G.A. Zavala[1], K. Prasad-Muliyala[2], F. Aslam[3], D. Barua[4], A. Haidar[4] C. Hewitt[1], R. Huque[4], S. Mansoor[3], P. Murthy[2], A. T. Nizami[3], N. Siddiqi[1,5,6], S. Sikander[7,8], K. Siddiqi[1,5], J.R. Boehnke[1,9] and on behalf of the IMPACT team.

Affiliations

[1]Department of Health Sciences, University of York, Heslington, YO10 5DD, York, UK

[2] National Institute of Mental Health and Neurosciences (NIMHANS), Bangalore, India.

[3] Institute of Psychiatry (IOP), Rawalpindi Medical University, Rawalpindi, Pakistan

[4]ARK Foundation, Dhaka, Bangladesh

[5] Hull York Medical School, University of York Communications Office, Heslington, York, UK

[6] Bradford District Care NHS Foundation Trust, Bradford, UK

[7] Human Development Research Foundation, Islamabad, Pakistan

[8]Public Health, Health Services Academy, Islamabad, Pakistan

[9]School of Health Sciences, University of Dundee, Dundee, UK

G.A. Zavala https://orcid.org/0000-0002-9825-8725 Corresponding author: gzavala@gmail.com

K. Prasad-Muliyala https://orcid.org/0000-0002-2580-9581

F. Aslam https://orcid.org/0000-0002-7847-7250

D. Barua https://orcid.org/0000-0002-0122-9048

A. Haidar https://orcid.org/0000-0001-9015-4414

C. Hewitt https://orcid.org/0000-0002-0415-3536

R. Huque http://orcid.org/0000-0003-4724-0581

S. Mansoor http://orcid.org://0000-0002-9837-6852

P. Murthy https://orcid.org/0000-0002-7738-9670

A. T. Nizami https://orcid.org/0000-0002-7853-8519

N. Siddiqi https://orcid.org/0000-0003-1794-2152

S. Sikander https://orcid.org/0000-0002-0223-7234

K. Siddiqi https://orcid.org/0000-0003-1529-7778

J.R. Boehnke https://orcid.org/0000-0003-0249-1870

For peer review only
For peer review only - http://bmjopen.bmj.com/site/about/guidelines.xhtml

Abstract

Introduction: People with severe mental illness (SMI) die on average 10-20 years earlier than the general population. Most of these deaths are due to physical health conditions. The aim of this cross-sectional study is to determine the prevalence of physical health conditions and their associations with health-risk behaviours, health-related quality of life and various demographic, behavioural, cognitive, psychological and social variables in people with SMI attending specialist mental health facilities in South Asia.

Methods and analysis: We will conduct a survey of patients with SMI attending specialist mental health facilities in Bangladesh, India and Pakistan (n=4,500). Diagnosis of SMI will be confirmed using the MINI V-6.0. We will collect information about: physical health and related health-risk behaviours (WHO STEPs); severity of common mental disorders (PHQ-9 and GAD-7); and health-related quality of life (EQ-5D-5L). We will measure blood pressure, height, weight and waist-circumference according to WHO guidelines. We will also measure glycated haemoglobin (HbA1c), lipid profile, thyroid function, liver function, creatinine and haemoglobin. Prevalence rates of physical health conditions and health-risk behaviours will be presented and compared with the WHO STEPs survey findings in the general population. Regression analyses will explore the association between health-risk behaviours, mental and physical health conditions.

Ethics and dissemination: The study has been approved by the ethics committees of the Department of Health Sciences University of York (UK), Centre for Injury Prevention and Rehabilitation (Bangladesh), Health Ministry Screening Committee and Indian Council of Medical Research (India) and National Bioethics Committee (Pakistan). Findings will be disseminated in peer reviewed articles, in local and international conferences and as reports for policy makers and stakeholders in the countries involved.

Trial registration: ISRCTN88485933; https://doi.org/10.1186/ISRCTN88485933, 3rd of June 2019

Key words: Multimorbidity, Comorbidity, Lifestyle, Severe mental illness, South Asia, health risk behaviours, schizophrenia, bipolar disorders, psychosis

For peer review only

Article summary

Strengths and limitations

- The study uses standardized tools to measure patient outcomes that will allow us to compare the findings with those of the general population.
- The survey will be conducted face to face by trained researchers
- The questionnaire has been translated into the most common languages used in Bangladesh India and Pakistan using validated translations of standardized measures where available.
- The survey is being conducted in specialist centres and is therefore a health care utilisation sample drawn largely from tertiary care.
- The study population may not be representative of all the people with severe mental illness in each country, as those not accessing specialist centres are not included in the survey.

For peer review only

For peer review only - http://bmjopen.bmj.com/site/about/guidelines.xhtml

Background

A considerable body of research has shown that people with severe mental illness (SMI; i.e. psychotic disorders, bipolar affective disorder and severe depression with psychotic symptoms) die on average 10-20 years earlier than the general population [1 2]. Around 80% of deaths in people with SMI are due to preventable physical illnesses, most commonly cardio-metabolic diseases, respiratory disorders, and infectious diseases [3 4].This excess mortality is a major global public health challenge but efforts to address it have been limited. Recent studies suggest that despite an overall improvement in life expectancy for both the general population and people with SMI, the absolute mortality gap between these two groups is actually widening [5-7].

Prevalence of most physical health conditions is higher and outcomes are poorer in this population[8]. Multimorbidity (the presence of two or more conditions) is also more common [9] in the presence of SMI, contributing to poorer physical health and quality of life. The majority of evidence for these health inequalities has been generated in high-income countries (HICs), but a small number of studies from low- and middle-income countries (LMICs) also show a similar pattern of increased mortality for people with SMI, with an even shorter life expectancy, and larger mortality gap [4 10-12]. The few studies available suggest that physical comorbidity in SMI is at least as prevalent as in developed countries [1].

In South Asia, rates of mental illness and physical non-communicable diseases have been increasing rapidly [13 14]. This increase, coupled with the lack of access to even basic mental healthcare [15], and neglect of the physical health needs of people with SMI by policymakers and healthcare services[16], means that the burden of disease due to physical disorders in people with mental illness is set to rise further, with a corresponding increase within country and global health inequalities. This increase is coupled with a recognized lack of information about the prevalence of physical conditions and health risk behaviours in the SMI population in South Asia [17]. This gap in knowledge, particularly in relation to health service planning in LMICs, has been pointed out repeatedly as a priority for research[18]. Addressing mental and physical health comorbidity in LMICs is also a global priority, recognized in global policies to help achieve the Sustainable Development Goals, including "ensuring healthy lives and promoting well-being for all" [19-21]. A detailed understanding of the prevalence of physical health conditions, health risk behaviours and health service utilization in SMI in LMICs is needed to progress this agenda[22].

Objectives

In people with SMI attending specialist mental health facilities:

1. Determine the prevalence of physical health conditions (i.e. type 2 diabetes, hypertension, heart disease, obesity and infectious diseases) and health-risk behaviours (i.e. diet, physical activity, alcohol and tobacco use) and compare these findings with those reported in the general population.
2. Determine the association between physical health conditions, health-risk behaviours, health-related quality of life and various demographic, behavioural, cognitive, psychological and social variables.
3. Identify health-service utilization and the type of lifestyle advice that has been offered for this SMI population.
4. Support clinical trial readiness by providing evidence-based findings regarding outcome measures and procedures relevant for clinical trials design.

Methods and analysis

Design, settings & population

We will conduct a cross-sectional survey among patients with a clinical diagnosis of SMI recruited at national specialist mental health institutions. In Bangladesh the study will be conducted at the National Institute of Mental Health and Hospital (NIMH) in Dhaka. NIMHH offers 200 beds for in-patient care and on average 400 out-patients attend every day. In India the study will be conducted at the National Institute of Mental Health and Neurosciences (NIMHANS) in Bengaluru, with 650 beds for in-patient care; on average at NIMHANS 400 out-patients attend every day. In Pakistan, the study will be

For peer review only

conducted at the Institute of Psychiatry (IOP) Rawalpindi Medical University, a WHO collaborating centre for the Eastern Mediterranean (EMRO). IOP offers 60 beds for inpatients and on average 400 out-patients attend every day. All three national institutes serve patients from all over the country. Adults (18 years and over) with a clinical diagnosis of SMI (defined by the International Classification of Disease 10th revision (ICD-10[23]) as schizophrenia, schizotypal and delusional disorders (F20-F29); bipolar affective disorder (F30, F31); and severe depression with psychotic symptoms (F32.3, F33.3))[23], who are able to provide informed consent as assessed by the treating clinician will be recruited. Patients who lack capacity to provide consent or are unable to complete study questionnaires will be excluded (figure 1). The data will be collected from June 2019 until September 2020.

Sample size

We aim to build as large a sample as possible within the resources available over the study period (n=1500 per country). As an indicative example of survey precision to address some of the key research questions, we provide a sample size estimate for investigating the prevalence of diabetes. Assuming a prevalence estimate of 10% (based on results from previous studies [24]), we will need 857 participants per country to achieve ± 2% precision assuming a 95% confidence interval[25].

Recruitment and random selection of the participants

Both in- and outpatients attending one of the three specialist mental health institutions collaborating in this study will be recruited. At NIMHH and NIMHANS patients will be selected randomly using random number tables generated centrally to determine for outpatients, which patient to approach on a given day, and for inpatients by randomly selecting beds. Recruitment is separated by in- vs outpatients, resulting in a proportion of 80% outpatients and 20% inpatients (which has been reported as the "usual" proportion of in- and outpatients). Due to the lower volume of patients and outpatients reaching the centre outside working hours at the IOP, all patients attending the IOP during the study duration will be invited to participate in the survey.

Informed consent

In the first instance, the researchers will give an information sheet to the patient, providing written and verbal information on the potential benefits and risks of taking part in the study and clarifying the assessments involved in the study. Only people who provided written informed consent will be included in the study. Where there are problems with literacy, the researchers will provide this information verbally to both the carer and the patient. Those unable to provide a signature will be requested to indicate their consent with a thumbprint. The information sheet will meet all the requirements of the relevant ethics committees. Maintenance of confidentiality and compliance with international, UK and relevant local Data Protection Acts will be emphasised to all study participants. Moreover, all participants will be informed that their participation in the study is voluntary, consent can be withdrawn at any stage and the decision about participation will not affect their care.

Mental capacity is time- and decision-specific. For patients who are assessed to lack capacity by their local physician, seeking informed consent will be delayed until capacity is regained, if feasible. No assessments will be continued where the patient appears reluctant, even where patient consent has been obtained.

Optional 'consent to contact' about future studies will be sought from survey participants. Permission will be sought to retain participant details and to contact participants about future studies that may be relevant for them. Again, it will be emphasised that this is voluntary, they can withdraw permission at any time, and this will not affect their care in any way. After consenting to participate, the participant will be given a unique patient identifier (ID) by the researcher. The patient consent and contact information will be entered into a database (participant log), which will be kept separate from the survey results database. The survey results database will include only the unique patient ID, and no patient identifiable data.

Confirmation of eligibility

SMI diagnosis will be confirmed by trained researchers using the relevant modules from the Mini-international neuropsychiatric interview (MINI) version 6.0 [26]. The MINI is a short diagnostic structured interview to explore mental disorders. It is designed to allow administration by non-specialist

For peer review only - http://bmjopen.bmj.com/site/about/guidelines.xhtml

interviewers[27]. The inter-rater reliability for the MINI will be assessed at each site at the start of study. Within each study site, at least 10 MINI questionnaires will be completed and compared between two different researchers blinded from each other. Where needed, further training will be offered to achieve acceptable inter-rater reliability before researchers are assigned to the study.

Data collection

We will conduct a face-to-face digital survey to collect information about physical health and health-risk behaviours, severity of common mental disorders and health-related quality of life using validated instruments as described below. The survey has been translated into the most common languages in the countries (Urdu in Pakistan, Bangla in Bangladesh and Hindi, Kannada, Tamil and Telugu for India). The team of interviewers in each county will include males and females and will include researchers that speak regional dialects, which is consistent with usual clinical practice in these settings [28].

The WHO STEPwise approach to Surveillance (STEPs) instrument Version 3.2 will be used to collect information about non-communicable diseases and related health-risk behaviours[29]. STEPs is an international standardized tool that will allow comparisons with the general population within the country and between countries[30]. The instrument has already been translated, used and validated in the general population in Bangladesh, India and Pakistan [31-33].

To allow patients to disclose information on sexually transmitted diseases, HIV diagnosis, alcohol and tobacco consumption, in agreement with patient's wishes, the interviewer will ask the caregivers/ attendants to leave the room for this part of the interview.

Demographic information

The STEPs demographic module will be used to collect information on age, sex, education, marital status, employment, household assets and income [29].

Mental health

We will collect information relevant to the SMI diagnosis of each participant, including duration of the mental illness, any treatment or medication and duration of the treatment and/or medication. The 9-item depression module (PHQ-9)[34] will be used to measure the severity of depressive symptoms and the generalized anxiety disorder 7 item scale (GAD-7)[35] for the severity of anxiety symptoms.

Health risk behaviours

The STEPs tobacco modules will be used to assess tobacco-related behaviours [29].

Alcohol consumption

The STEPs alcohol module will be used to determine lifetime abstainers, past 12 months abstainers and current users of alcohol using the WHO cut-off scores [29].

Diet

The STEPs diet module will be used to record the number of days that respondents consumed fruit and vegetables in a typical week, and the number of servings of fruit and vegetables consumed on average per day [36].

Physical activity

The STEPs physical activity module will be used to record activity for transport purposes (e.g. walking, cycling), vigorous and moderate activity at work, and vigorous, moderate activity in leisure time, and time spent sitting. Show-cards with culturally relevant examples will be used to aid respondents in classifying activities. Analysis and categorization will follow the WHO guidelines[37].

Non-communicable diseases

Any medically-diagnosed history of raised blood pressure, heart disease, hypercholesterolemia, and high blood glucose, and treatment advised by a health worker for these diseases (such as prescribed medicines, a special diet, advice to reduce salt intake, lose weight, stop smoking, or do more exercise) will be collected using the STEPs module for non-communicable diseases. Lung disease is not included

For peer review only - http://bmjopen.bmj.com/site/about/guidelines.xhtml

in the STEPSs survey. However, it will be recorded asking the same set of questions as the non-communicable diseases included in the STEPs survey.

Infectious diseases

Self-report of medically diagnosed history of hepatitis B, C, syphilis, tuberculosis, HIV and intestinal parasites will be recorded.

Risk behaviours related to sexually transmitted diseases

Risk behaviours related to sexually transmitted diseases will be assessed using three questions that examine the presence of multiple sexual partners, unprotected sexual contact and use of injectable drugs that have been adapted from the 10 item HIV risk Screening Instrument [38]. This instrument has been previously used in research involving persons with severe mental illness in India [39].

Health-Related Quality of life

The EQ-5D-5L will be used to measure health-related quality of life [40 41]. The EQ-5D-5L is a standardised measure of health status, it provides a simple, generic measure of health for clinical and economic appraisal. The impact of disease/disorder is characterised on five dimensions (mobility, self-care, ability to undertake usual activities, pain, anxiety / depression). The EQ-5D-5L further assesses a patient's subjective evaluation of their health state based on a visual analogue scale (EQ-5d-VAS) between "0 - worst imaginable health state" and "100 - best imaginable health state". We will use validated translations provided by EUROQOL.

Blood pressure and heart rate

Blood pressure (BP) and pulse rate will be taken three times using an automated blood pressure measuring instrument (OMRON®). For BP measurement the participant must be comfortable and rested. If he/she had been exerting themselves, then there will be a minimum of a 15 minute rest period before the recording is taken. There should be at least a three minute gap between the BP recordings. The procedure will follow the step wise instructions in the WHO STEPS surveillance manual[42].

Height weight and waist circumference

Height will be measured to a precision of 0.1 cm using a portable height measuring board without footwear and headgear. Weight will be measured in kilograms using a portable weighing scale placed on a firm flat surface with light clothing and without footwear and socks. Waist circumference will be measured in to a precision of 0.1 cm, using a flexible fiberglass anthropometric tape at the end of normal expiration, at the midpoint between the lower margin of the last palpable rib and the top of the iliac crest (hip bone), with the arms relaxed at the sides. All measurements will be taken in duplicate and the average of the two values will be recorded and the protocols of the WHO STEPS surveillance manual will be followed[42].

Biochemical analysis

A blood sample (8 to 9 ml) will be taken from consenting participants for: glycated haemoglobin (HbA1c), lipid profile (triglycerides, total cholesterol, HDL cholesterol, LDL cholesterol), thyroid function tests (includes T3, T4, TSH), liver function tests (total Bilirubin, AST, ALT, ALP, total protein, albumin, albumin to globulin ratio), creatinine and haemoglobin.

Data management

To ensure quality of collected data, validation, checking, proofing and cleaning procedures will be carried out according to standard procedures[43]. All consent forms will be stored separately from survey data in locked cabinets in locked offices at study research offices at each study site. All coded data will be transferred to, and stored as anonymous data at the University of York which will act as the data curator. A secure password protected and encrypted electronic database will be set up to store the data.

For peer review only - http://bmjopen.bmj.com/site/about/guidelines.xhtml

Statistical analysis

Quantitative data will be summarised using descriptive statistics. First, prevalence rates of infectious diseases, long-term physical health conditions (e.g. diabetes, heart disease) and health-risk behaviours for type II diabetes, cardiovascular and respiratory disorders (e.g. tobacco use, obesity, physical activity, diet) will be estimated in the severe mental illness population. Second, we will examine the associations between physical health conditions and health-risk behaviours, using regression models with random intercepts for data collection site with the following potential covariates: demographic variables (i.e. age, sex, and level of education), history of mental and physical conditions and biomarkers (e.g. BMI, blood pressure). These regression models will also be used to calculate prevalence rates of physical health conditions and health-risk behaviours adjusted for these covariates (e.g., for comparison with external general population data) Further regression analyses will explore the association between mental and physical health conditions and health-related quality of life measured using EQ-5D-5L. Regression models will take account of the nature and distribution of data (e.g. count versus continuous data; skewed versus normally distributed data).To investigate the prevalence of physical health conditions and health risk behaviours in the general population, we will extract aggregated data on the prevalence of physical health conditions, health risk behaviours and lifestyle advice from the last available WHO STEPs survey from Bangladesh[44], India[45] and Pakistan[46].

Ethics and dissemination

The study has been approved by the ethics committees of the Department of Health Sciences University of York (UK), Centre for Injury Prevention and Research (Bangladesh), Health Ministry Screening Committee and Indian Council of Medical Research (India) and National Bioethics Committee (Pakistan).

The study will adhere to the fundamental principles of human rights and dignity laid down in the Declaration of Helsinki[47]. Study procedures will comply with legislation and guidance for good practice governing the participation in research of people lacking capacity as set out in the Mental Capacity Act (UK) 2005[48]. Participants will not receive any financial inducement to participate, but the results of the physical measurements and blood tests will be shared with the participant and the treating clinician.

As a non-interventional study, there is minimal risk of adverse events associated with the study. Due to the vulnerability of the severe mental illness population, however, there is a potential (albeit low) risk that some questions or the burden of the assessments may cause distress for participants or the family carers. If there is any indication of this, the survey or assessments will be stopped, the participant offered reassurance and support, and if needed, referral to the clinical team. If a participant during the interview reveals any suicidal ideation, the interviewer will refer them to the treating clinician for a clinical risk assessment and further management. If a physical condition or blood test abnormality (outside the normal range for age and sex) is detected as a part of the research assessments, the research team will inform the clinician responsible for the patient, who will evaluate the result and give the patient the option of referral to a specialist if this is required for their condition.

Community, patient and public involvement

A 'Community Panel' comprising stakeholders from these constituencies ensures community, patient and public involvement. The Panel has reviewed the planned survey and advised on its feasibility. The panel will continue to provide feedback on the conduct of the study and presentation of findings. To support dissemination efforts, findings will be presented to the community panel, with advice sought about the format and content of lay summaries and other outputs aimed at patients and the public.

Dissemination

Findings will be disseminated in peer-reviewed articles, in local and international conferences and as reports for policy makers and stakeholders in the countries involved. An open access repository of all materials will be built that covers the survey materials as well as statistical syntax used by the team publications.

Discussion

For peer review only - http://bmjopen.bmj.com/site/about/guidelines.xhtml

People with SMI have a reduced life expectancy. Research in high income countries has shown that a large proportion of this inequality is accounted for by chronic diseases [1 2]. This has been consistently observed in high income countries [3 4]. However information is lacking in minority groups and LMICs, which is surprising considering the increased risk of physical health conditions in these populations due to poverty, education, poor diet, low access to healthcare, exposure to infectious diseases and other environmental factors [49]. The IMPACT SMI survey will address this gap in knowledge, demonstrating the prevalence of different physical health conditions and health-risk behaviours in the SMI population, and the association of each of these conditions in terms of health related quality of life. It will support SMI clinical trial readiness by providing evidence-based findings regarding outcome measures and procedures relevant for clinical trials design.

Strengths and limitations

To the best of the authors' knowledge, this will be the first large scale study to investigate the physical health and health-risk behaviours in the SMI population in LMICs. The main limitation is that the survey is conducted in specialist centres (with patients with capacity) and is therefore a health care utilisation sample drawn largely from tertiary care. The gaps in treatment and care utilisation and systematic factors influencing these have been well documented [50], therefore, the study population may not be representative of the total severe mental illness population in each country. However, a community survey would not be feasible, and primary and secondary care mental health services are not sufficiently developed to be used to recruit participants for this survey (see for example work by PRIME [15] and Emerald [51] programmes on strengthening these services as access points). However, unlike services in high-income countries, tertiary care services in these South Asian countries accept self-referral without the need for primary or secondary care referral and we include outpatient services offered by the collaborating centres which often function as 'the first port of call' for people with mental illness[52-54]. Therefore, the selected centres are likely covering a broad range of the treatment-seeking population. We will not have a control group since the study is only conducted in people with SMI, however we are using the same questions and measurements as the WHO STEPs survey. This will allow us to compare the prevalence of physical health conditions and health risk behaviours with that of a survey representative for the population in each country.

Conclusion

Multimorbidity in people with SMI is not well documented particularly in low- and middle-income countries. The IMPACT survey will be the first to study it using a standardised approach in South Asia that can be compared to the general population.

Acknowledgements

We would like to acknowledge Dr Sue Bellass, Prof. Santosh Kumar Chaturvedi, Dr Arun Kandasamy, Dr Noreen Mdege, Dr Masuma Mishu, Dr Emily Peckham, Prof. Simon Gilbody and the research teams at ARK foundation and The National Institute of Mental Health (NIMH) in Bangladesh , Institute of Psychiatry in Pakistan (IOP) and The National Institute of Mental Health and Neuro-Sciences (NIMHANS) in India for their contribution to the study. We want to thank all the participants and caregivers for taking the time to complete the survey.

References

1. Liu NH, Daumit GL, Dua T, et al. Excess mortality in persons with severe mental disorders: a multilevel intervention framework and priorities for clinical practice, policy and research agendas. *World psychiatry : official journal of the World Psychiatric Association (WPA)* 2017;16(1):30-40. doi: 10.1002/wps.20384 [published Online First: 2017/01/28]
2. Walker ER, McGee RE, Druss BG. Mortality in mental disorders and global disease burden implications: a systematic review and meta-analysis. *JAMA psychiatry* 2015;72(4):334-41. doi: 10.1001/jamapsychiatry.2014.2502 [published Online First: 2015/02/12]
3. Laursen TM. Life expectancy among persons with schizophrenia or bipolar affective disorder. *Schizophrenia research* 2011;131(1-3):101-4. doi: 10.1016/j.schres.2011.06.008 [published Online First: 2011/07/12]
4. Fekadu A, Medhin G, Kebede D, et al. Excess mortality in severe mental illness: 10-year population-based cohort study in rural Ethiopia. *The British journal of psychiatry : the journal of mental*

For peer review only - http://bmjopen.bmj.com/site/about/guidelines.xhtml

science 2015;206(4):289-96. doi: 10.1192/bjp.bp.114.149112 [published Online First: 2015/02/07]

5. Olfson M, Gerhard T, Huang C, et al. Premature Mortality Among Adults With Schizophrenia in the United States. *JAMA psychiatry* 2015;72(12):1172-81. doi: 10.1001/jamapsychiatry.2015.1737 [published Online First: 2015/10/29]
6. Saha S, Chant D, McGrath J. A systematic review of mortality in schizophrenia: is the differential mortality gap worsening over time? *Archives of general psychiatry* 2007;64(10):1123-31. doi: 10.1001/archpsyc.64.10.1123 [published Online First: 2007/10/03]
7. Chesney E, Goodwin GM, Fazel S. Risks of all-cause and suicide mortality in mental disorders: a meta-review. *World psychiatry : official journal of the World Psychiatric Association (WPA)* 2014;13(2):153-60. doi: 10.1002/wps.20128 [published Online First: 2014/06/04]
8. Mai Q, Holman CD, Sanfilippo FM, et al. The impact of mental illness on potentially preventable hospitalisations: a population-based cohort study. *BMC psychiatry* 2011;11:163. doi: 10.1186/1471-244X-11-163 [published Online First: 2011/10/12]
9. Reilly S, Olier I, Planner C, et al. Inequalities in physical comorbidity: a longitudinal comparative cohort study of people with severe mental illness in the UK. *BMJ open* 2015;5(12):e009010. doi: 10.1136/bmjopen-2015-009010 [published Online First: 2015/12/17]
10. Kurihara T, Kato M, Reverger R, et al. Seventeen-year clinical outcome of schizophrenia in Bali. *European psychiatry : the journal of the Association of European Psychiatrists* 2011;26(5):333-8. doi: 10.1016/j.eurpsy.2011.04.003 [published Online First: 2011/06/24]
11. Mogga S, Prince M, Alem A, et al. Outcome of major depression in Ethiopia: population-based study. *The British journal of psychiatry : the journal of mental science* 2006;189:241-6. doi: 10.1192/bjp.bp.105.013417 [published Online First: 2006/09/02]
12. Teferra S, Shibre T, Fekadu A, et al. Five-year mortality in a cohort of people with schizophrenia in Ethiopia. *BMC psychiatry* 2011;11:165. doi: 10.1186/1471-244x-11-165 [published Online First: 2011/10/12]
13. Nambiar D, Razzak J, Afsana K, et al. Mental illness and injuries: emerging health challenges of urbanisation in South Asia. *bmj* 2017;357:j1126.
14. Misra A, Tandon N, Ebrahim S, et al. Diabetes, cardiovascular disease, and chronic kidney disease in South Asia: current status and future directions. *bmj* 2017;357:j1420.
15. Lund C, Tomlinson M, Patel V. Integration of mental health into primary care in low-and middle-income countries: the PRIME mental healthcare plans. *The British Journal of Psychiatry* 2016;208(s56):s1-s3.
16. Shavitt S, Cho YI, Johnson TP, et al. Culture moderates the relation between perceived stress, social support, and mental and physical health. *Journal of Cross-Cultural Psychology* 2016;47(7):956-80.
17. The L. Making more of multimorbidity: an emerging priority. *Lancet (London, England)* 2018;391(10131):1637.
18. Hanlon C. Next steps for meeting the needs of people with severe mental illness in low-and middle-income countries. *Epidemiology and psychiatric sciences* 2017;26(4):348-54.
19. Collins PY, Insel TR, Chockalingam A, et al. Grand challenges in global mental health: integration in research, policy, and practice. *PLoS Med* 2013;10(4):e1001434. doi: 10.1371/journal.pmed.1001434 [published Online First: 2013/05/03]
20. Baingana F, al'Absi M, Becker AE, et al. Global research challenges and opportunities for mental health and substance-use disorders. *Nature* 2015;527(7578):S172-7. doi: 10.1038/nature16032 [published Online First: 2015/11/19]
21. UN. Transforming our world: the 2030 Agenda for Sustainable Development. New York: United Nations, 2015.
22. Stubbs B, Koyanagi A, Veronese N, et al. Physical multimorbidity and psychosis: comprehensive cross sectional analysis including 242,952 people across 48 low-and middle-income countries. *BMC medicine* 2016;14(1):189.
23. Organization WH. The ICD-10 classification of mental and behavioural disorders: diagnostic criteria for research: World Health Organization 1993.
24. Vancampfort D, Correll CU, Galling B, et al. Diabetes mellitus in people with schizophrenia, bipolar disorder and major depressive disorder: a systematic review and large scale meta-analysis. *World psychiatry : official journal of the World Psychiatric Association (WPA)* 2016;15(2):166-74. doi: 10.1002/wps.20309 [published Online First: 2016/06/07]

For peer review only - http://bmjopen.bmj.com/site/about/guidelines.xhtml

25. Buderer NMF. Statistical methodology: I. Incorporating the prevalence of disease into the sample size calculation for sensitivity and specificity. *Academic Emergency Medicine* 1996;3(9):895-900.
26. Lecrubier Y, Sheehan DV, Weiller E, et al. The Mini International Neuropsychiatric Interview (MINI). A short diagnostic structured interview: reliability and validity according to the CIDI. *European Psychiatry* 1997;12(5):224-31. doi: https://doi.org/10.1016/S0924-9338(97)83296-8
27. Sheehan DV, Lecrubier Y, Harnett Sheehan K, et al. The validity of the Mini International Neuropsychiatric Interview (MINI) according to the SCID-P and its reliability. *European Psychiatry* 1997;12(5):232-41. doi: https://doi.org/10.1016/S0924-9338(97)83297-X
28. Chand P, Murthy P, Arunachalam V, et al. Service utilization in a tertiary psychiatric care setting in South India. *Asian journal of psychiatry* 2010;3(4):222-26.
29. Bonita R, Winkelmann R, Douglas KA, et al. The WHO Stepwise approach to surveillance (STEPS) of non-communicable disease risk factors. Global behavioral risk factor surveillance: Springer 2003:9-22.
30. Bonita R, De Courten M, Dwyer T, et al. Surveillance of risk factors for noncommunicable diseases: the WHO STEPwise approach: summary: Noncommunicable Diseases and Mental Health, World Health Organization, 2001.
31. Nishtar S, Bile KM, Ahmed A, et al. Integrated population-based surveillance of noncommunicable diseases: the Pakistan model. *American journal of preventive medicine* 2005;29(5):102-06.
32. Mohan V, Mathur P, Deepa R, et al. Urban rural differences in prevalence of self-reported diabetes in India—The WHO–ICMR Indian NCD risk factor surveillance. *Diabetes research and clinical practice* 2008;80(1):159-68.
33. Zaman MM, Rahman MM, Rahman MR, et al. Prevalence of risk factors for non-communicable diseases in Bangladesh: results from STEPS survey 2010. *Indian J Public Health* 2016;60(1):17-25.
34. Kroenke K, Spitzer RL. The PHQ-9: a new depression diagnostic and severity measure. *Psychiatric annals* 2002;32(9):509-15.
35. Löwe B, Decker O, Müller S, et al. Validation and standardization of the Generalized Anxiety Disorder Screener (GAD-7) in the general population. *Medical care* 2008;46(3):266-74.
36. Fulton SL, McKinley MC, Young IS, et al. The effect of increasing fruit and vegetable consumption on overall diet: a systematic review and meta-analysis. *Critical reviews in food science and nutrition* 2016;56(5):802-16.
37. Cleland CL, Hunter RF, Kee F, et al. Validity of the global physical activity questionnaire (GPAQ) in assessing levels and change in moderate-vigorous physical activity and sedentary behaviour. *BMC public health* 2014;14(1):1255.
38. Gerbert B, Bronstone A, McPhee S, et al. Development and testing of an HIV-risk screening instrument for use in health care settings. *American Journal of Preventive Medicine* 1998;15(2):103-13.
39. Chandra PS, Carey MP, Carey KB, et al. HIV risk behaviour among psychiatric inpatients: results from a hospital-wide screening study in southern India. *International journal of STD & AIDS* 2003;14(8):532-38.
40. Hurst NP, Kind P, Ruta D, et al. Measuring health-related quality of life in rheumatoid arthritis: validity, responsiveness and reliability of EuroQol (EQ-5D). *British journal of rheumatology* 1997;36(5):551-9.
41. EuroQol Group. EQ-5D-5L User Guide: Basic information on how to use the EQ-5D-5L instrument (version 2.0) 2013 04/03/2015. 1990. http://www.euroqol.org/fileadmin/user_upload/Documenten/PDF/Folders_Flyers/UserGuide_EQ-5D-5L_v2.0_October_2013.pdf. (accessed 09/05/2017).
42. Organization WH. WHO STEPS surveillance manual: the WHO STEPwise approach to chronic disease risk factor surveillance: Geneva: World Health Organization, 2005.
43. Corti L, Van den Eynden V, Bishop L, et al. Managing and sharing research data: a guide to good practice: Sage 2014.
44. Zaman MM, Bhuiyan MR, Karim MN, et al. Clustering of non-communicable diseases risk factors in Bangladeshi adults: an analysis of STEPS survey 2013. *BMC public health* 2015;15(1):659.
45. Bhattacherjee S, Datta S, Roy JK, et al. A cross-sectional assessment of risk factors of non-communicable diseases in a sub-Himalayan region of West Bengal, India using who steps approach. *Journal of The Association of Physicians of India* 2015;63:34.

For peer review only - http://bmjopen.bmj.com/site/about/guidelines.xhtml

46. Rafique I, Saqib MA, Munir MA, et al. Prevalence of risk factors for noncommunicable diseases in adults: key findings from the Pakistan STEPS survey. *East Mediterr Health J* 2018;24(1):33-41.
47. Williams JR. The Declaration of Helsinki and public health. *Bulletin of the World Health Organization* 2008;86:650-52.
48. Boyle G. The Mental Capacity Act 2005: promoting the citizenship of people with dementia? *Health & social care in the community* 2008;16(5):529-37.
49. Allen L, Williams J, Townsend N, et al. Socioeconomic status and non-communicable disease behavioural risk factors in low-income and lower-middle-income countries: a systematic review. *The Lancet Global Health* 2017;5(3):e277-e89.
50. Evans-Lacko S, Aguilar-Gaxiola S, Al-Hamzawi A, et al. Socio-economic variations in the mental health treatment gap for people with anxiety, mood, and substance use disorders: results from the WHO World Mental Health (WMH) surveys. *Psychol Med* 2018;48(9):1560-71. doi: 10.1017/s0033291717003336 [published Online First: 2017/11/28]
51. Semrau M, Evans-Lacko S, Alem A, et al. Strengthening mental health systems in low-and middle-income countries: the Emerald programme. *BMC medicine* 2015;13(1):79.
52. Afsar HA, Younus M. Patient referral at the grass-roots level in Pakistan. *Nat Sci(Internet)* 2004;2(4):18-27.
53. Karim S, Saeed K, Rana MH, et al. Pakistan mental health country profile. *International Review of Psychiatry* 2004;16(1-2):83-92.
54. Organization WH. Bangladesh health system review: Manila: WHO Regional Office for the Western Pacific 2015.

Authors' contributions

NS conceived the study, KP, FA, DB, RH, SM, PM, AN, SK and KS provided important contextual information, KP, FP, DB, RH, SM, NS and JB developed the methodology and selected the outcomes, GZ, AH, KH and JB developed the analysis plan, GZ, NS, KS and JB wrote the manuscript, and all authors revised and approved the manuscript.

Funding statement

This research was funded by the National Institute for Health Research (NIHR) (17/63/130) using UK aid from the UK Government to support global health research. The views expressed in this publication are those of the author(s) and not necessarily those of the NIHR or the UK Department of Health and Social Care. The funders had no role in study design, data collection and analysis, decision to publish, or preparation of the manuscript.

Competing interests statement

The authors declare that they have no competing interests

Word count

3159 words

For peer review only - http://bmjopen.bmj.com/site/about/guidelines.xhtml

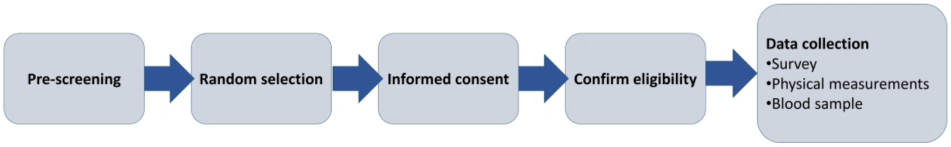

Figure 1. Flow diagram of the study

1399x600mm (96 x 96 DPI)

INVESTIGATING MENTAL AND PHYSICAL COMORBIDITY: Protocol for a survey in people with severe mental illness in South Asia (IMPACT SMI survey)

STROBE Statement—Checklist of items that should be included in reports of ***cross-sectional studies***

	Item No	Recommendation
**Title and abstract**	1	(*a*) Indicate the study's design with a commonly used term in the title or the abstract **Title and abstract lines 1 to 56**
		(*b*) Provide in the abstract an informative and balanced summary of what was done and what was found **Abstract lines 32 to 56**
**Introduction**		
Background/rationale	2	Explain the scientific background and rationale for the investigation being reported **Background paragraphs 1 to 3**
Objectives	3	State specific objectives, including any prespecified hypotheses **Background, objectives section lines 93 to 101**
**Methods**		
Study design	4	Present key elements of study design early in the paper **Methods paragraph 1 design setting and population 103 to 115**
Setting	5	Describe the setting, locations, and relevant dates, including periods of recruitment, exposure, follow-up, and data collection **Methods paragraph 1 design setting and population lines 103 to 115**
Participants	6	(*a*) Give the eligibility criteria, and the sources and methods of selection of participants **Methods paragraph 1 design setting and population lines 103 to 115**
Variables	7	Clearly define all outcomes, exposures, predictors, potential confounders, and effect modifiers. Give diagnostic criteria, if applicable **Methods, data collection section lines 158 to 231**
Data sources/ measurement	8*	For each variable of interest, give sources of data and details of methods of assessment (measurement). Describe comparability of assessment methods if there is more than one group **Methods, data collection section lines 158 to 231**
Bias	9	Describe any efforts to address potential sources of bias **Discussion, strengths and limitation section lines 297 to 307**
Study size	10	Explain how the study size was arrived at **Methods section Sample size lines 298 to 311**
Quantitative variables	11	Explain how quantitative variables were handled in the analyses. If applicable, describe which groupings were chosen and why **NA**
Statistical methods	12	(*a*) Describe all statistical methods, including those used to control for confounding **Methods Statistical analysis section lines 240 to 255**
		(*b*) Describe any methods used to examine subgroups and interactions **Methods Statistical analysis section lines 240 to 255**
		(*c*) Explain how missing data were addressed **NA**
		(*d*) If applicable, describe analytical methods taking account of sampling strategy **Methods, Recruitment and random selection of the participants lines 123 to 126**
		(*e*) Describe any sensitivity analyses

For peer review only - http://bmjopen.bmj.com/site/about/guidelines.xhtml

		**Methods Statistical analysis section lines 239 to 255**
**Results**		
Participants	13*	(a) Report numbers of individuals at each stage of study—eg numbers potentially eligible, examined for eligibility, confirmed eligible, included in the study, completing follow-up, and analysed<br>**NA**
		(b) Give reasons for non-participation at each stage<br>NA
		(c) Consider use of a flow diagram<br>**NA**
Descriptive data	14*	(a) Give characteristics of study participants (eg demographic, clinical, social) and information on exposures and potential confounders
		(b) Indicate number of participants with missing data for each variable of interest<br>**NA**
Outcome data	15*	Report numbers of outcome events or summary measures<br>**NA**
Main results	16	(*a*) Give unadjusted estimates and, if applicable, confounder-adjusted estimates and their precision (eg, 95% confidence interval). Make clear which confounders were adjusted for and why they were included
		(*b*) Report category boundaries when continuous variables were categorized
		(b) If relevant, consider translating estimates of relative risk into absolute risk for a meaningful time period<br>**NA**
Other analyses	17	Report other analyses done—eg analyses of subgroups and interactions, and sensitivity analyses<br>**NA**
**Discussion**		
Key results	18	Summarise key results with reference to study objectives<br>**NA**
Limitations	19	Discuss limitations of the study, taking into account sources of potential bias or imprecision. Discuss both direction and magnitude of any potential bias<br>**NA**
Interpretation	20	Give a cautious overall interpretation of results considering objectives, limitations, multiplicity of analyses, results from similar studies, and other relevant evidence<br>**NA**
Generalisability	21	Discuss the generalisability (external validity) of the study results<br>**NA**
**Other information**		
Funding	22	Give the source of funding and the role of the funders for the present study and, if applicable, for the original study on which the present article is based<br>**NA**

*Give information separately for exposed and unexposed groups.

For peer review only - http://bmjopen.bmj.com/site/about/guidelines.xhtml

Note: An Explanation and Elaboration article discusses each checklist item and gives methodological background and published examples of transparent reporting. The STROBE checklist is best used in conjunction with this article (freely available on the Web sites of PLoS Medicine at http://www.plosmedicine.org/, Annals of Internal Medicine at http://www.annals.org/, and Epidemiology at http://www.epidem.com/). Information on the STROBE Initiative is available at www.strobe-statement.org.

For peer review only

For peer review only - http://bmjopen.bmj.com/site/about/guidelines.xhtml

BMJ Open

Prevalence of physical health conditions and health risk behaviours in people with severe mental Illness in South Asia: Protocol for a cross-sectional study (IMPACT SMI survey)

Journal:	*BMJ Open*
Manuscript ID	bmjopen-2020-037869.R2
Article Type:	Protocol
Date Submitted by the Author:	18-Aug-2020
Complete List of Authors:	Zavala, Gerardo A.; University of York,<br>Prasad, Krishna; National Institute of Mental Health and Neuro Sciences<br>Aslam, Faiza; Institute of Psychiatry<br>Barua, Deepa; ARK Foundation<br>Haidar, Asiful; ARK Foundation<br>Hewitt, Catherine; The University of York, Health Sciences<br>Huque, Rumana; ARK Foundation<br>Mansoor, Sonia; Institute of Psychiatry<br>Murthy, Pratima; National Institute of Mental Health and NeuroScineces, Centre for Addiction Medicine<br>Nizami, Asad; Institute of Psychiatry<br>Siddiqi, Najma; University of York, Psychiatry, Hull York Medical School, York and Bradford District Care NHS Foundation Trust, Bradford, UK<br>Sikander, Siham; Health Services Academy<br>Siddiqi, Kamran; University of York, UK, Institute of Health Sciences<br>Boehnke, Jan Rasmus; University of Dundee, School of Nursing and Health Sciences
**Primary Subject Heading**:	Global health
Secondary Subject Heading:	Global health, Mental health, Epidemiology, Public health
Keywords:	MENTAL HEALTH, Schizophrenia & psychotic disorders < PSYCHIATRY, PSYCHIATRY

SCHOLARONE™
Manuscripts

I, the Submitting Author has the right to grant and does grant on behalf of all authors of the Work (as defined in the below author licence), an exclusive licence and/or a non-exclusive licence for contributions from authors who are: i) UK Crown employees; ii) where BMJ has agreed a CC-BY licence shall apply, and/or iii) in accordance with the terms applicable for US Federal Government officers or employees acting as part of their official duties; on a worldwide, perpetual, irrevocable, royalty-free basis to BMJ Publishing Group Ltd ("BMJ") its licensees and where the relevant Journal is co-owned by BMJ to the co-owners of the Journal, to publish the Work in this journal and any other BMJ products and to exploit all rights, as set out in our licence.

The Submitting Author accepts and understands that any supply made under these terms is made by BMJ to the Submitting Author unless you are acting as an employee on behalf of your employer or a postgraduate student of an affiliated institution which is paying any applicable article publishing charge ("APC") for Open Access articles. Where the Submitting Author wishes to make the Work available on an Open Access basis (and intends to pay the relevant APC), the terms of reuse of such Open Access shall be governed by a Creative Commons licence – details of these licences and which Creative Commons licence will apply to this Work are set out in our licence referred to above.

Other than as permitted in any relevant BMJ Author's Self Archiving Policies, I confirm this Work has not been accepted for publication elsewhere, is not being considered for publication elsewhere and does not duplicate material already published. I confirm all authors consent to publication of this Work and authorise the granting of this licence.

For peer review only

Prevalence of physical health conditions and health risk behaviours in people with severe mental Illness in South Asia: Protocol for a cross-sectional study (IMPACT SMI survey)

G.A. Zavala[1], K. Prasad-Muliyala[2], F. Aslam[3], D. Barua[4], A. Haidar[4] C. Hewitt[1], R. Huque[4], S. Mansoor[3], P. Murthy[2], A. T. Nizami[3], N. Siddiqi[1,5,6], S. Sikander[7,8], K. Siddiqi[1,5], J.R. Boehnke[1,9] and on behalf of the IMPACT team.

Affiliations

[1]Department of Health Sciences, University of York, Heslington, YO10 5DD, York, UK

[2] National Institute of Mental Health and Neurosciences (NIMHANS), Bangalore, India.

[3] Institute of Psychiatry (IOP), Rawalpindi Medical University, Rawalpindi, Pakistan

[4]ARK Foundation, Dhaka, Bangladesh

[5] Hull York Medical School, University of York Communications Office, Heslington, York, UK

[6] Bradford District Care NHS Foundation Trust, Bradford, UK

[7] Human Development Research Foundation, Islamabad, Pakistan

[8]Public Health, Health Services Academy, Islamabad, Pakistan

[9]School of Health Sciences, University of Dundee, Dundee, UK

G.A. Zavala https://orcid.org/0000-0002-9825-8725 Corresponding author: gzavala@gmail.com

K. Prasad-Muliyala https://orcid.org/0000-0002-2580-9581

F. Aslam https://orcid.org/0000-0002-7847-7250

D. Barua https://orcid.org/0000-0002-0122-9048

A. Haidar https://orcid.org/0000-0001-9015-4414

C. Hewitt https://orcid.org/0000-0002-0415-3536

R. Huque http://orcid.org/0000-0003-4724-0581

S. Mansoor http://orcid.org://0000-0002-9837-6852

P. Murthy https://orcid.org/0000-0002-7738-9670

A. T. Nizami https://orcid.org/0000-0002-7853-8519

N. Siddiqi https://orcid.org/0000-0003-1794-2152

S. Sikander https://orcid.org/0000-0002-0223-7234

K. Siddiqi https://orcid.org/0000-0003-1529-7778

J.R. Boehnke https://orcid.org/0000-0003-0249-1870

For peer review only

For peer review only - http://bmjopen.bmj.com/site/about/guidelines.xhtml

Abstract

Introduction: People with severe mental illness (SMI) die on average 10-20 years earlier than the general population. Most of these deaths are due to physical health conditions. The aim of this cross-sectional study is to determine the prevalence of physical health conditions and their associations with health-risk behaviours, health-related quality of life and various demographic, behavioural, cognitive, psychological and social variables in people with SMI attending specialist mental health facilities in South Asia.

Methods and analysis: We will conduct a survey of patients with SMI attending specialist mental health facilities in Bangladesh, India and Pakistan (n=4,500). Diagnosis of SMI will be confirmed using the MINI V-6.0. We will collect information about: physical health and related health-risk behaviours (WHO STEPs); severity of common mental disorders (PHQ-9 and GAD-7); and health-related quality of life (EQ-5D-5L). We will measure blood pressure, height, weight and waist-circumference according to WHO guidelines. We will also measure glycated haemoglobin (HbA1c), lipid profile, thyroid function, liver function, creatinine and haemoglobin. Prevalence rates of physical health conditions and health-risk behaviours will be presented and compared with the WHO STEPs survey findings in the general population. Regression analyses will explore the association between health-risk behaviours, mental and physical health conditions.

Ethics and dissemination: The study has been approved by the ethics committees of the Department of Health Sciences University of York (UK), Centre for Injury Prevention and Rehabilitation (Bangladesh), Health Ministry Screening Committee and Indian Council of Medical Research (India) and National Bioethics Committee (Pakistan). Findings will be disseminated in peer reviewed articles, in local and international conferences and as reports for policy makers and stakeholders in the countries involved.

Trial registration: ISRCTN88485933; https://doi.org/10.1186/ISRCTN88485933, 3rd of June 2019

Key words: Multimorbidity, Comorbidity, Lifestyle, Severe mental illness, South Asia, health risk behaviours, schizophrenia, bipolar disorders, psychosis

For peer review only

Article summary

Strengths and limitations

- The study uses standardized tools to measure patient outcomes that will allow us to compare the findings with those of the general population.
- The survey will be conducted face to face by trained researchers
- The questionnaire has been translated into the most common languages used in Bangladesh India and Pakistan using validated translations of standardized measures where available.
- The survey is being conducted in specialist centres and is therefore a health care utilisation sample drawn largely from tertiary care.
- The study population may not be representative of all the people with severe mental illness in each country, as those not accessing specialist centres are not included in the survey.

For peer review only

For peer review only - http://bmjopen.bmj.com/site/about/guidelines.xhtml

Background

A considerable body of research has shown that people with severe mental illness (SMI; i.e. psychotic disorders, bipolar affective disorder and severe depression with psychotic symptoms) die on average 10-20 years earlier than the general population [1 2]. Around 80% of deaths in people with SMI are due to preventable physical illnesses, most commonly cardio-metabolic diseases, respiratory disorders, and infectious diseases [3 4].This excess mortality is a major global public health challenge but efforts to address it have been limited. Recent studies suggest that despite an overall improvement in life expectancy for both the general population and people with SMI, the absolute mortality gap between these two groups is actually widening [5-7].

Prevalence of most physical health conditions is higher and outcomes are poorer in this population[8]. Multimorbidity (the presence of two or more conditions) is also more common [9] in the presence of SMI, contributing to poorer physical health and quality of life. The majority of evidence for these health inequalities has been generated in high-income countries (HICs), but a small number of studies from low- and middle-income countries (LMICs) also show a similar pattern of increased mortality for people with SMI, with an even shorter life expectancy, and larger mortality gap [4 10-12]. The few studies available suggest that physical comorbidity in SMI is at least as prevalent as in developed countries [1].

In South Asia, rates of mental illness and physical non-communicable diseases have been increasing rapidly [13 14]. This increase, coupled with the lack of access to even basic mental healthcare [15], and neglect of the physical health needs of people with SMI by policymakers and healthcare services[16], means that the burden of disease due to physical disorders in people with mental illness is set to rise further, with a corresponding increase within country and global health inequalities. This increase is coupled with a recognized lack of information about the prevalence of physical conditions and health risk behaviours in the SMI population in South Asia [17]. This gap in knowledge, particularly in relation to health service planning in LMICs, has been pointed out repeatedly as a priority for research[18]. Addressing mental and physical health comorbidity in LMICs is also a global priority, recognized in global policies to help achieve the Sustainable Development Goals, including "ensuring healthy lives and promoting well-being for all" [19-21]. A detailed understanding of the prevalence of physical health conditions, health risk behaviours and health service utilization in SMI in LMICs is needed to progress this agenda[22].

Objectives

In people with SMI attending specialist mental health facilities:

1. Determine the prevalence of physical health conditions (i.e. type 2 diabetes, hypertension, heart disease, obesity and infectious diseases) and health-risk behaviours (i.e. diet, physical activity, alcohol and tobacco use) and compare these findings with those reported in the general population.
2. Determine the association between physical health conditions, health-risk behaviours, health-related quality of life and various demographic, behavioural, cognitive, psychological and social variables.
3. Identify health-service utilization and the type of lifestyle advice that has been offered for this SMI population.
4. Support clinical trial readiness by providing evidence-based findings regarding outcome measures and procedures relevant for clinical trials design.

Methods and analysis

Design, settings & population

We will conduct a cross-sectional survey among patients with a clinical diagnosis of SMI recruited at national specialist mental health institutions. In Bangladesh the study will be conducted at the National Institute of Mental Health and Hospital (NIMH) in Dhaka. NIMHH offers 200 beds for in-patient care and on average 400 out-patients attend every day. In India the study will be conducted at the National Institute of Mental Health and Neurosciences (NIMHANS) in Bengaluru, with 650 beds for in-patient care; on average at NIMHANS 400 out-patients attend every day. In Pakistan, the study will be

For peer review only - http://bmjopen.bmj.com/site/about/guidelines.xhtml

conducted at the Institute of Psychiatry (IOP) Rawalpindi Medical University, a WHO collaborating centre for the Eastern Mediterranean (EMRO). IOP offers 60 beds for inpatients and on average 400 out-patients attend every day. All three national institutes serve patients from all over the country. Adults (18 years and over) with a clinical diagnosis of SMI (defined by the International Classification of Disease 10th revision (ICD-10[23]) as schizophrenia, schizotypal and delusional disorders (F20-F29); bipolar affective disorder (F30, F31); and severe depression with psychotic symptoms (F32.3, F33.3))[23], who are able to provide informed consent as assessed by the treating clinician will be recruited. Patients who lack capacity to provide consent or are unable to complete study questionnaires will be excluded (figure 1). The data will be collected from June 2019 until September 2020.

Sample size

We aim to build as large a sample as possible within the resources available over the study period (n=1500 per country). As an indicative example of survey precision to address some of the key research questions, we provide a sample size estimate for investigating the prevalence of diabetes. Assuming a prevalence estimate of 10% (based on results from previous studies [24]), we will need 857 participants per country to achieve ± 2% precision assuming a 95% confidence interval[25].

Recruitment and random selection of the participants

Both in- and outpatients attending one of the three specialist mental health institutions collaborating in this study will be recruited. At NIMHH and NIMHANS patients will be selected randomly using random number tables generated centrally to determine for outpatients, which patient to approach on a given day, and for inpatients by randomly selecting beds. Recruitment is separated by in- vs outpatients, resulting in a proportion of 80% outpatients and 20% inpatients (which has been reported as the "usual" proportion of in- and outpatients). Due to the lower volume of patients and outpatients reaching the centre outside working hours at the IOP, all patients attending the IOP during the study duration will be invited to participate in the survey.

Informed consent

In the first instance, the researchers will give an information sheet to the patient, providing written and verbal information on the potential benefits and risks of taking part in the study and clarifying the assessments involved in the study. Only people who provided written informed consent will be included in the study. Where there are problems with literacy, the researchers will provide this information verbally to both the carer and the patient. Those unable to provide a signature will be requested to indicate their consent with a thumbprint. The information sheet will meet all the requirements of the relevant ethics committees. Maintenance of confidentiality and compliance with international, UK and relevant local Data Protection Acts will be emphasised to all study participants. Moreover, all participants will be informed that their participation in the study is voluntary, consent can be withdrawn at any stage and the decision about participation will not affect their care.

Mental capacity is time- and decision-specific. For patients who are assessed to lack capacity by their local physician, seeking informed consent will be delayed until capacity is regained, if feasible. No assessments will be continued where the patient appears reluctant, even where patient consent has been obtained.

Optional 'consent to contact' about future studies will be sought from survey participants. Permission will be sought to retain participant details and to contact participants about future studies that may be relevant for them. Again, it will be emphasised that this is voluntary, they can withdraw permission at any time, and this will not affect their care in any way. After consenting to participate, the participant will be given a unique patient identifier (ID) by the researcher. The patient consent and contact information will be entered into a database (participant log), which will be kept separate from the survey results database. The survey results database will include only the unique patient ID, and no patient identifiable data.

Confirmation of eligibility

SMI diagnosis will be confirmed by trained researchers using the relevant modules from the Mini-international neuropsychiatric interview (MINI) version 6.0 [26]. The MINI is a short diagnostic structured interview to explore mental disorders. It is designed to allow administration by non-specialist

For peer review only - http://bmjopen.bmj.com/site/about/guidelines.xhtml

interviewers[27]. The inter-rater reliability for the MINI will be assessed at each site at the start of study. Within each study site, at least 10 MINI questionnaires will be completed and compared between two different researchers blinded from each other. Where needed, further training will be offered to achieve acceptable inter-rater reliability before researchers are assigned to the study.

Data collection

We will conduct a face-to-face digital survey to collect information about physical health and health-risk behaviours, severity of common mental disorders and health-related quality of life using validated instruments as described below. The survey has been translated into the most common languages in the countries (Urdu in Pakistan, Bangla in Bangladesh and Hindi, Kannada, Tamil and Telugu for India). The team of interviewers in each county will include males and females and will include researchers that speak regional dialects, which is consistent with usual clinical practice in these settings [28]. Participants will be able to express a preference to be interviewed by a researcher of the same gender, to reduce response bias that might arise due to the gender of the interviewer.

The WHO STEPwise approach to Surveillance (STEPs) instrument Version 3.2 will be used to collect information about non-communicable diseases and related health-risk behaviours[29]. STEPs is an international standardized tool that will allow comparisons with the general population within the country and between countries[30]. The instrument has already been translated, used and validated in the general population in Bangladesh, India and Pakistan [31-33].

To allow patients to disclose information on sexually transmitted diseases, HIV diagnosis, alcohol and tobacco consumption, in agreement with patient's wishes, the interviewer will ask the caregivers/ attendants to leave the room for this part of the interview.

Demographic information

The STEPs demographic module will be used to collect information on age, sex, education, marital status, employment, household assets and income [29].

Mental health

We will collect information relevant to the SMI diagnosis of each participant, including duration of the mental illness, any treatment or medication and duration of the treatment and/or medication. The 9-item depression module (PHQ-9)[34] will be used to measure the severity of depressive symptoms and the generalized anxiety disorder 7 item scale (GAD-7)[35] for the severity of anxiety symptoms.

Health risk behaviours

The STEPs tobacco modules will be used to assess tobacco-related behaviours [29].

Alcohol consumption

The STEPs alcohol module will be used to determine lifetime abstainers, past 12 months abstainers and current users of alcohol using the WHO cut-off scores [29].

Diet

The STEPs diet module will be used to record the number of days that respondents consumed fruit and vegetables in a typical week, and the number of servings of fruit and vegetables consumed on average per day [36].

Physical activity

The STEPs physical activity module will be used to record activity for transport purposes (e.g. walking, cycling), vigorous and moderate activity at work, and vigorous, moderate activity in leisure time, and time spent sitting. Show-cards with culturally relevant examples will be used to aid respondents in classifying activities. Analysis and categorization will follow the WHO guidelines[37].

Non-communicable diseases

Any medically-diagnosed history of raised blood pressure, heart disease, hypercholesterolemia, and high blood glucose, and treatment advised by a health worker for these diseases (such as prescribed medicines, a special diet, advice to reduce salt intake, lose weight, stop smoking, or do more exercise)

For peer review only - http://bmjopen.bmj.com/site/about/guidelines.xhtml

will be collected using the STEPs module for non-communicable diseases. Lung disease is not included in the STEPSs survey. However, it will be recorded asking the same set of questions as the non-communicable diseases included in the STEPs survey.

Infectious diseases

Self-report of medically diagnosed history of hepatitis B, C, syphilis, tuberculosis, HIV and intestinal parasites will be recorded.

Risk behaviours related to sexually transmitted diseases

Risk behaviours related to sexually transmitted diseases will be assessed using three questions that examine the presence of multiple sexual partners, unprotected sexual contact and use of injectable drugs that have been adapted from the 10 item HIV risk Screening Instrument [38]. This instrument has been previously used in research involving persons with severe mental illness in India [39].

Health-Related Quality of life

The EQ-5D-5L will be used to measure health-related quality of life [40 41]. The EQ-5D-5L is a standardised measure of health status, it provides a simple, generic measure of health for clinical and economic appraisal. The impact of disease/disorder is characterised on five dimensions (mobility, self-care, ability to undertake usual activities, pain, anxiety / depression). The EQ-5D-5L further assesses a patient's subjective evaluation of their health state based on a visual analogue scale (EQ-5d-VAS) between "0 - worst imaginable health state" and "100 - best imaginable health state". We will use validated translations provided by EUROQOL.

Blood pressure and heart rate

Blood pressure (BP) and pulse rate will be taken three times using an automated blood pressure measuring instrument (OMRON®). For BP measurement the participant must be comfortable and rested. If he/she had been exerting themselves, then there will be a minimum of a 15 minute rest period before the recording is taken. There should be at least a three minute gap between the BP recordings. The procedure will follow the step wise instructions in the WHO STEPS surveillance manual[42].

Height weight and waist circumference

Height will be measured to a precision of 0.1 cm using a portable height measuring board without footwear and headgear. Weight will be measured in kilograms using a portable weighing scale placed on a firm flat surface with light clothing and without footwear and socks. Waist circumference will be measured in to a precision of 0.1 cm, using a flexible fiberglass anthropometric tape at the end of normal expiration, at the midpoint between the lower margin of the last palpable rib and the top of the iliac crest (hip bone), with the arms relaxed at the sides. All measurements will be taken in duplicate and the average of the two values will be recorded and the protocols of the WHO STEPS surveillance manual will be followed[42].

Biochemical analysis

A blood sample (8 to 9 ml) will be taken from consenting participants for: glycated haemoglobin (HbA1c), lipid profile (triglycerides, total cholesterol, HDL cholesterol, LDL cholesterol), thyroid function tests (includes T3, T4, TSH), liver function tests (total Bilirubin, AST, ALT, ALP, total protein, albumin, albumin to globulin ratio), creatinine and haemoglobin.

Data management

To ensure quality of collected data, validation, checking, proofing and cleaning procedures will be carried out according to standard procedures[43]. All consent forms will be stored separately from survey data in locked cabinets in locked offices at study research offices at each study site. All coded data will be transferred to, and stored as anonymous data at the University of York which will act as the data curator. A secure password protected and encrypted electronic database will be set up to store the data.

For peer review only - http://bmjopen.bmj.com/site/about/guidelines.xhtml

Statistical analysis

Quantitative data will be summarised using descriptive statistics. First, prevalence rates of infectious diseases, long-term physical health conditions (e.g. diabetes, heart disease) and health-risk behaviours for type II diabetes, cardiovascular and respiratory disorders (e.g. tobacco use, obesity, physical activity, diet) will be estimated in the severe mental illness population. Second, we will examine the associations between physical health conditions and health-risk behaviours, using regression models with random intercepts for data collection site with the following potential covariates: demographic variables (i.e. age, sex, and level of education), history of mental and physical conditions and biomarkers (e.g. BMI, blood pressure). These regression models will also be used to calculate prevalence rates of physical health conditions and health-risk behaviours adjusted for these covariates (e.g., for comparison with external general population data) Further regression analyses will explore the association between mental and physical health conditions and health-related quality of life measured using EQ-5D-5L. Regression models will take account of the nature and distribution of data (e.g. count versus continuous data; skewed versus normally distributed data).To investigate the prevalence of physical health conditions and health risk behaviours in the general population, we will extract aggregated data on the prevalence of physical health conditions, health risk behaviours and lifestyle advice from the last available WHO STEPs survey from Bangladesh[44], India[45] and Pakistan[46].

Ethics and dissemination

The study has been approved by the ethics committees of the Department of Health Sciences University of York (UK), Centre for Injury Prevention and Research (Bangladesh), Health Ministry Screening Committee and Indian Council of Medical Research (India) and National Bioethics Committee (Pakistan).

The study will adhere to the fundamental principles of human rights and dignity laid down in the Declaration of Helsinki[47]. Study procedures will comply with legislation and guidance for good practice governing the participation in research of people lacking capacity as set out in the Mental Capacity Act (UK) 2005[48]. Participants will not receive any financial inducement to participate, but the results of the physical measurements and blood tests will be shared with the participant and the treating clinician.

As a non-interventional study, there is minimal risk of adverse events associated with the study. Due to the vulnerability of the severe mental illness population, however, there is a potential (albeit low) risk that some questions or the burden of the assessments may cause distress for participants or the family carers. If there is any indication of this, the survey or assessments will be stopped, the participant offered reassurance and support, and if needed, referral to the clinical team. If a participant during the interview reveals any suicidal ideation, the interviewer will refer them to the treating clinician for a clinical risk assessment and further management. If a physical condition or blood test abnormality (outside the normal range for age and sex) is detected as a part of the research assessments, the research team will inform the clinician responsible for the patient, who will evaluate the result and give the patient the option of referral to a specialist if this is required for their condition.

Community, patient and public involvement

A 'Community Panel' comprising stakeholders from these constituencies ensures community, patient and public involvement. The Panel has reviewed the planned survey and advised on its feasibility. The panel will continue to provide feedback on the conduct of the study and presentation of findings. To support dissemination efforts, findings will be presented to the community panel, with advice sought about the format and content of lay summaries and other outputs aimed at patients and the public.

Dissemination

Findings will be disseminated in peer-reviewed articles, in local and international conferences and as reports for policy makers and stakeholders in the countries involved. An open access repository of all materials will be built that covers the survey materials as well as statistical syntax used by the team publications.

Discussion

For peer review only - http://bmjopen.bmj.com/site/about/guidelines.xhtml

People with SMI have a reduced life expectancy. Research in high income countries has shown that a large proportion of this inequality is accounted for by chronic diseases [1 2]. This has been consistently observed in high income countries [3 4]. However information is lacking in minority groups and LMICs, which is surprising considering the increased risk of physical health conditions in these populations due to poverty, education, poor diet, low access to healthcare, exposure to infectious diseases and other environmental factors [49]. The IMPACT SMI survey will address this gap in knowledge, demonstrating the prevalence of different physical health conditions and health-risk behaviours in the SMI population, and the association of each of these conditions in terms of health related quality of life. It will support SMI clinical trial readiness by providing evidence-based findings regarding outcome measures and procedures relevant for clinical trials design.

Strengths and limitations

To the best of the authors' knowledge, this will be the first large scale study to investigate the physical health and health-risk behaviours in the SMI population in LMICs. The main limitation is that the survey is conducted in specialist centres (with patients with capacity) and is therefore a health care utilisation sample drawn largely from tertiary care. The gaps in treatment and care utilisation and systematic factors influencing these have been well documented [50], therefore, the study population may not be representative of the total severe mental illness population in each country. However, a community survey would not be feasible, and primary and secondary care mental health services are not sufficiently developed to be used to recruit participants for this survey (see for example work by PRIME [15] and Emerald [51] programmes on strengthening these services as access points). However, unlike services in high-income countries, tertiary care services in these South Asian countries accept self-referral without the need for primary or secondary care referral and we include outpatient services offered by the collaborating centres which often function as 'the first port of call' for people with mental illness[52-54]. Therefore, the selected centres are likely covering a broad range of the treatment-seeking population. We will not have a control group since the study is only conducted in people with SMI, however we are using the same questions and measurements as the WHO STEPs survey. This will allow us to compare the prevalence of physical health conditions and health risk behaviours with that of a survey representative for the population in each country.

Acknowledgements

We would like to acknowledge Dr Sue Bellass, Prof. Santosh Kumar Chaturvedi, Dr Arun Kandasamy, Dr Noreen Mdege, Dr Masuma Mishu, Dr Emily Peckham, Prof. Simon Gilbody and the research teams at ARK foundation and The National Institute of Mental Health (NIMH) in Bangladesh , Institute of Psychiatry in Pakistan (IOP) and The National Institute of Mental Health and Neuro-Sciences (NIMHANS) in India for their contribution to the study. We want to thank all the participants and caregivers for taking the time to complete the survey.

References

1. Liu NH, Daumit GL, Dua T, et al. Excess mortality in persons with severe mental disorders: a multilevel intervention framework and priorities for clinical practice, policy and research agendas. *World psychiatry : official journal of the World Psychiatric Association (WPA)* 2017;16(1):30-40. doi: 10.1002/wps.20384 [published Online First: 2017/01/28]
2. Walker ER, McGee RE, Druss BG. Mortality in mental disorders and global disease burden implications: a systematic review and meta-analysis. *JAMA psychiatry* 2015;72(4):334-41. doi: 10.1001/jamapsychiatry.2014.2502 [published Online First: 2015/02/12]
3. Laursen TM. Life expectancy among persons with schizophrenia or bipolar affective disorder. *Schizophrenia research* 2011;131(1-3):101-4. doi: 10.1016/j.schres.2011.06.008 [published Online First: 2011/07/12]
4. Fekadu A, Medhin G, Kebede D, et al. Excess mortality in severe mental illness: 10-year population-based cohort study in rural Ethiopia. *The British journal of psychiatry : the journal of mental science* 2015;206(4):289-96. doi: 10.1192/bjp.bp.114.149112 [published Online First: 2015/02/07]
5. Olfson M, Gerhard T, Huang C, et al. Premature Mortality Among Adults With Schizophrenia in the United States. *JAMA psychiatry* 2015;72(12):1172-81. doi: 10.1001/jamapsychiatry.2015.1737 [published Online First: 2015/10/29]

For peer review only - http://bmjopen.bmj.com/site/about/guidelines.xhtml

6. Saha S, Chant D, McGrath J. A systematic review of mortality in schizophrenia: is the differential mortality gap worsening over time? *Archives of general psychiatry* 2007;64(10):1123-31. doi: 10.1001/archpsyc.64.10.1123 [published Online First: 2007/10/03]
7. Chesney E, Goodwin GM, Fazel S. Risks of all-cause and suicide mortality in mental disorders: a meta-review. *World psychiatry : official journal of the World Psychiatric Association (WPA)* 2014;13(2):153-60. doi: 10.1002/wps.20128 [published Online First: 2014/06/04]
8. Mai Q, Holman CD, Sanfilippo FM, et al. The impact of mental illness on potentially preventable hospitalisations: a population-based cohort study. *BMC psychiatry* 2011;11:163. doi: 10.1186/1471-244X-11-163 [published Online First: 2011/10/12]
9. Reilly S, Olier I, Planner C, et al. Inequalities in physical comorbidity: a longitudinal comparative cohort study of people with severe mental illness in the UK. *BMJ open* 2015;5(12):e009010. doi: 10.1136/bmjopen-2015-009010 [published Online First: 2015/12/17]
10. Kurihara T, Kato M, Reverger R, et al. Seventeen-year clinical outcome of schizophrenia in Bali. *European psychiatry : the journal of the Association of European Psychiatrists* 2011;26(5):333-8. doi: 10.1016/j.eurpsy.2011.04.003 [published Online First: 2011/06/24]
11. Mogga S, Prince M, Alem A, et al. Outcome of major depression in Ethiopia: population-based study. *The British journal of psychiatry : the journal of mental science* 2006;189:241-6. doi: 10.1192/bjp.bp.105.013417 [published Online First: 2006/09/02]
12. Teferra S, Shibre T, Fekadu A, et al. Five-year mortality in a cohort of people with schizophrenia in Ethiopia. *BMC psychiatry* 2011;11:165. doi: 10.1186/1471-244x-11-165 [published Online First: 2011/10/12]
13. Nambiar D, Razzak J, Afsana K, et al. Mental illness and injuries: emerging health challenges of urbanisation in South Asia. *bmj* 2017;357:j1126.
14. Misra A, Tandon N, Ebrahim S, et al. Diabetes, cardiovascular disease, and chronic kidney disease in South Asia: current status and future directions. *bmj* 2017;357:j1420.
15. Lund C, Tomlinson M, Patel V. Integration of mental health into primary care in low-and middle-income countries: the PRIME mental healthcare plans. *The British Journal of Psychiatry* 2016;208(s56):s1-s3.
16. Shavitt S, Cho YI, Johnson TP, et al. Culture moderates the relation between perceived stress, social support, and mental and physical health. *Journal of Cross-Cultural Psychology* 2016;47(7):956-80.
17. The L. Making more of multimorbidity: an emerging priority. *Lancet (London, England)* 2018;391(10131):1637.
18. Hanlon C. Next steps for meeting the needs of people with severe mental illness in low-and middle-income countries. *Epidemiology and psychiatric sciences* 2017;26(4):348-54.
19. Collins PY, Insel TR, Chockalingam A, et al. Grand challenges in global mental health: integration in research, policy, and practice. *PLoS Med* 2013;10(4):e1001434. doi: 10.1371/journal.pmed.1001434 [published Online First: 2013/05/03]
20. Baingana F, al'Absi M, Becker AE, et al. Global research challenges and opportunities for mental health and substance-use disorders. *Nature* 2015;527(7578):S172-7. doi: 10.1038/nature16032 [published Online First: 2015/11/19]
21. UN. Transforming our world: the 2030 Agenda for Sustainable Development. New York: United Nations, 2015.
22. Stubbs B, Koyanagi A, Veronese N, et al. Physical multimorbidity and psychosis: comprehensive cross sectional analysis including 242,952 people across 48 low-and middle-income countries. *BMC medicine* 2016;14(1):189.
23. Organization WH. The ICD-10 classification of mental and behavioural disorders: diagnostic criteria for research: World Health Organization 1993.
24. Vancampfort D, Correll CU, Galling B, et al. Diabetes mellitus in people with schizophrenia, bipolar disorder and major depressive disorder: a systematic review and large scale meta-analysis. *World psychiatry : official journal of the World Psychiatric Association (WPA)* 2016;15(2):166-74. doi: 10.1002/wps.20309 [published Online First: 2016/06/07]
25. Buderer NMF. Statistical methodology: I. Incorporating the prevalence of disease into the sample size calculation for sensitivity and specificity. *Academic Emergency Medicine* 1996;3(9):895-900.

For peer review only

For peer review only - http://bmjopen.bmj.com/site/about/guidelines.xhtml

26. Lecrubier Y, Sheehan DV, Weiller E, et al. The Mini International Neuropsychiatric Interview (MINI). A short diagnostic structured interview: reliability and validity according to the CIDI. *European Psychiatry* 1997;12(5):224-31. doi: https://doi.org/10.1016/S0924-9338(97)83296-8
27. Sheehan DV, Lecrubier Y, Harnett Sheehan K, et al. The validity of the Mini International Neuropsychiatric Interview (MINI) according to the SCID-P and its reliability. *European Psychiatry* 1997;12(5):232-41. doi: https://doi.org/10.1016/S0924-9338(97)83297-X
28. Chand P, Murthy P, Arunachalam V, et al. Service utilization in a tertiary psychiatric care setting in South India. *Asian journal of psychiatry* 2010;3(4):222-26.
29. Bonita R, Winkelmann R, Douglas KA, et al. The WHO Stepwise approach to surveillance (STEPS) of non-communicable disease risk factors. Global behavioral risk factor surveillance: Springer 2003:9-22.
30. Bonita R, De Courten M, Dwyer T, et al. Surveillance of risk factors for noncommunicable diseases: the WHO STEPwise approach: summary: Noncommunicable Diseases and Mental Health, World Health Organization, 2001.
31. Nishtar S, Bile KM, Ahmed A, et al. Integrated population-based surveillance of noncommunicable diseases: the Pakistan model. *American journal of preventive medicine* 2005;29(5):102-06.
32. Mohan V, Mathur P, Deepa R, et al. Urban rural differences in prevalence of self-reported diabetes in India—The WHO–ICMR Indian NCD risk factor surveillance. *Diabetes research and clinical practice* 2008;80(1):159-68.
33. Zaman MM, Rahman MM, Rahman MR, et al. Prevalence of risk factors for non-communicable diseases in Bangladesh: results from STEPS survey 2010. *Indian J Public Health* 2016;60(1):17-25.
34. Kroenke K, Spitzer RL. The PHQ-9: a new depression diagnostic and severity measure. *Psychiatric annals* 2002;32(9):509-15.
35. Löwe B, Decker O, Müller S, et al. Validation and standardization of the Generalized Anxiety Disorder Screener (GAD-7) in the general population. *Medical care* 2008;46(3):266-74.
36. Fulton SL, McKinley MC, Young IS, et al. The effect of increasing fruit and vegetable consumption on overall diet: a systematic review and meta-analysis. *Critical reviews in food science and nutrition* 2016;56(5):802-16.
37. Cleland CL, Hunter RF, Kee F, et al. Validity of the global physical activity questionnaire (GPAQ) in assessing levels and change in moderate-vigorous physical activity and sedentary behaviour. *BMC public health* 2014;14(1):1255.
38. Gerbert B, Bronstone A, McPhee S, et al. Development and testing of an HIV-risk screening instrument for use in health care settings. *American Journal of Preventive Medicine* 1998;15(2):103-13.
39. Chandra PS, Carey MP, Carey KB, et al. HIV risk behaviour among psychiatric inpatients: results from a hospital-wide screening study in southern India. *International journal of STD & AIDS* 2003;14(8):532-38.
40. Hurst NP, Kind P, Ruta D, et al. Measuring health-related quality of life in rheumatoid arthritis: validity, responsiveness and reliability of EuroQol (EQ-5D). *British journal of rheumatology* 1997;36(5):551-9.
41. EuroQol Group. EQ-5D-5L User Guide: Basic information on how to use the EQ-5D-5L instrument (version 2.0) 2013 04/03/2015. 1990. http://www.euroqol.org/fileadmin/user_upload/Documenten/PDF/Folders_Flyers/UserGuide_EQ-5D-5L_v2.0_October_2013.pdf. (accessed 09/05/2017).
42. Organization WH. WHO STEPS surveillance manual: the WHO STEPwise approach to chronic disease risk factor surveillance: Geneva: World Health Organization, 2005.
43. Corti L, Van den Eynden V, Bishop L, et al. Managing and sharing research data: a guide to good practice: Sage 2014.
44. Zaman MM, Bhuiyan MR, Karim MN, et al. Clustering of non-communicable diseases risk factors in Bangladeshi adults: an analysis of STEPS survey 2013. *BMC public health* 2015;15(1):659.
45. Bhattacherjee S, Datta S, Roy JK, et al. A cross-sectional assessment of risk factors of non-communicable diseases in a sub-Himalayan region of West Bengal, India using who steps approach. *Journal of The Association of Physicians of India* 2015;63:34.
46. Rafique I, Saqib MA, Munir MA, et al. Prevalence of risk factors for noncommunicable diseases in adults: key findings from the Pakistan STEPS survey. *East Mediterr Health J* 2018;24(1):33-41.

For peer review only - http://bmjopen.bmj.com/site/about/guidelines.xhtml

47. Williams JR. The Declaration of Helsinki and public health. *Bulletin of the World Health Organization* 2008;86:650-52.
48. Boyle G. The Mental Capacity Act 2005: promoting the citizenship of people with dementia? *Health & social care in the community* 2008;16(5):529-37.
49. Allen L, Williams J, Townsend N, et al. Socioeconomic status and non-communicable disease behavioural risk factors in low-income and lower-middle-income countries: a systematic review. *The Lancet Global Health* 2017;5(3):e277-e89.
50. Evans-Lacko S, Aguilar-Gaxiola S, Al-Hamzawi A, et al. Socio-economic variations in the mental health treatment gap for people with anxiety, mood, and substance use disorders: results from the WHO World Mental Health (WMH) surveys. *Psychol Med* 2018;48(9):1560-71. doi: 10.1017/s0033291717003336 [published Online First: 2017/11/28]
51. Semrau M, Evans-Lacko S, Alem A, et al. Strengthening mental health systems in low-and middle-income countries: the Emerald programme. *BMC medicine* 2015;13(1):79.
52. Afsar HA, Younus M. Patient referral at the grass-roots level in Pakistan. *Nat Sci(Internet)* 2004;2(4):18-27.
53. Karim S, Saeed K, Rana MH, et al. Pakistan mental health country profile. *International Review of Psychiatry* 2004;16(1-2):83-92.
54. Organization WH. Bangladesh health system review: Manila: WHO Regional Office for the Western Pacific 2015.

Authors' contributions

NS conceived the study, KP, FA, DB, RH, SM, PM, AN, SS and KS provided important contextual information, KP, FA, DB, AH, SM, NS, KS and JB developed the methodology and selected the outcomes, GZ, AH, CH and JB developed the analysis plan, GZ, NS, KS and JB wrote the manuscript, and all authors revised and approved the manuscript.

Funding statement

This research was funded by the National Institute for Health Research (NIHR) (17/63/130) using UK aid from the UK Government to support global health research. The views expressed in this publication are those of the author(s) and not necessarily those of the NIHR or the UK Department of Health and Social Care. The funders had no role in study design, data collection and analysis, decision to publish, or preparation of the manuscript.

Competing interests statement

The authors declare that they have no competing interests

Word count

3159 words

Figure legend

Figure 1. Study flow chart

For peer review only - http://bmjopen.bmj.com/site/about/guidelines.xhtml

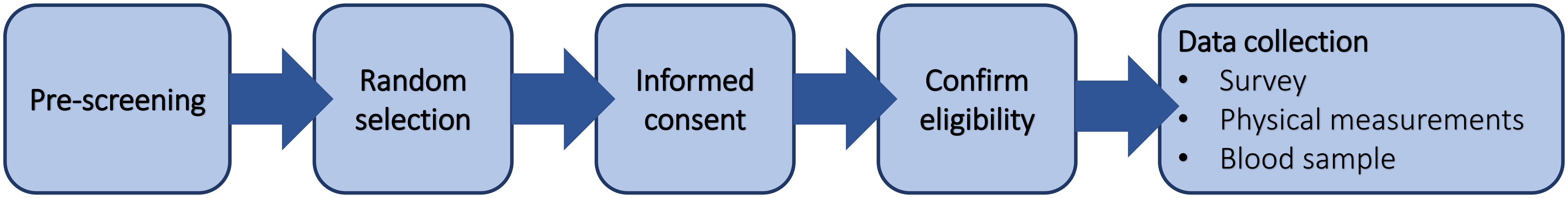
Pre-screening
Random selection
Informed consent
Confirm eligibility
Data collection
• Survey
• Physical measurements
• Blood sample

INVESTIGATING MENTAL AND PHYSICAL COMORBIDITY: Protocol for a survey in people with severe mental illness in South Asia (IMPACT SMI survey)

STROBE Statement—Checklist of items that should be included in reports of ***cross-sectional studies***

	Item No	Recommendation
**Title and abstract**	1	(*a*) Indicate the study's design with a commonly used term in the title or the abstract<br>**Title and abstract lines 1 to 56**
		(*b*) Provide in the abstract an informative and balanced summary of what was done and what was found<br>**Abstract lines 32 to 56**
**Introduction**		
Background/rationale	2	Explain the scientific background and rationale for the investigation being reported<br>**Background paragraphs 1 to 3**
Objectives	3	State specific objectives, including any prespecified hypotheses<br>**Background, objectives section lines 93 to 101**
**Methods**		
Study design	4	Present key elements of study design early in the paper<br>**Methods paragraph 1 design setting and population 103 to 115**
Setting	5	Describe the setting, locations, and relevant dates, including periods of recruitment, exposure, follow-up, and data collection<br>**Methods paragraph 1 design setting and population lines 103 to 115**
Participants	6	(*a*) Give the eligibility criteria, and the sources and methods of selection of participants<br>**Methods paragraph 1 design setting and population lines 103 to 115**
Variables	7	Clearly define all outcomes, exposures, predictors, potential confounders, and effect modifiers. Give diagnostic criteria, if applicable<br>**Methods, data collection section lines 158 to 231**
Data sources/ measurement	8*	For each variable of interest, give sources of data and details of methods of assessment (measurement). Describe comparability of assessment methods if there is more than one group<br>**Methods, data collection section lines 158 to 231**
Bias	9	Describe any efforts to address potential sources of bias<br>**Discussion, strengths and limitation section lines 297 to 307**
Study size	10	Explain how the study size was arrived at<br>**Methods section Sample size lines 298 to 311**
Quantitative variables	11	Explain how quantitative variables were handled in the analyses. If applicable, describe which groupings were chosen and why<br>**NA**
Statistical methods	12	(*a*) Describe all statistical methods, including those used to control for confounding<br>**Methods Statistical analysis section lines 240 to 255**
		(*b*) Describe any methods used to examine subgroups and interactions<br>**Methods Statistical analysis section lines 240 to 255**
		(*c*) Explain how missing data were addressed<br>**NA**
		(*d*) If applicable, describe analytical methods taking account of sampling strategy<br>**Methods, Recruitment and random selection of the participants lines 123 to 126**
		(*e*) Describe any sensitivity analyses

For peer review only - http://bmjopen.bmj.com/site/about/guidelines.xhtml

		**Methods Statistical analysis section lines 239 to 255**
**Results**		
Participants	13*	(a) Report numbers of individuals at each stage of study—eg numbers potentially eligible, examined for eligibility, confirmed eligible, included in the study, completing follow-up, and analysed<br>**NA**
		(b) Give reasons for non-participation at each stage<br>NA
		(c) Consider use of a flow diagram<br>**NA**
Descriptive data	14*	(a) Give characteristics of study participants (eg demographic, clinical, social) and information on exposures and potential confounders
		(b) Indicate number of participants with missing data for each variable of interest<br>**NA**
Outcome data	15*	Report numbers of outcome events or summary measures<br>**NA**
Main results	16	(*a*) Give unadjusted estimates and, if applicable, confounder-adjusted estimates and their precision (eg, 95% confidence interval). Make clear which confounders were adjusted for and why they were included
		(*b*) Report category boundaries when continuous variables were categorized
		(b) If relevant, consider translating estimates of relative risk into absolute risk for a meaningful time period<br>**NA**
Other analyses	17	Report other analyses done—eg analyses of subgroups and interactions, and sensitivity analyses<br>**NA**
**Discussion**		
Key results	18	Summarise key results with reference to study objectives<br>**NA**
Limitations	19	Discuss limitations of the study, taking into account sources of potential bias or imprecision. Discuss both direction and magnitude of any potential bias<br>**NA**
Interpretation	20	Give a cautious overall interpretation of results considering objectives, limitations, multiplicity of analyses, results from similar studies, and other relevant evidence<br>**NA**
Generalisability	21	Discuss the generalisability (external validity) of the study results<br>**NA**
**Other information**		
Funding	22	Give the source of funding and the role of the funders for the present study and, if applicable, for the original study on which the present article is based<br>**NA**

*Give information separately for exposed and unexposed groups.

For peer review only

Note: An Explanation and Elaboration article discusses each checklist item and gives methodological background and published examples of transparent reporting. The STROBE checklist is best used in conjunction with this article (freely available on the Web sites of PLoS Medicine at http://www.plosmedicine.org/, Annals of Internal Medicine at http://www.annals.org/, and Epidemiology at http://www.epidem.com/). Information on the STROBE Initiative is available at www.strobe-statement.org.

For peer review only

For peer review only - http://bmjopen.bmj.com/site/about/guidelines.xhtml
